# Inflammatory and cardiovascular diseases biomarkers in chronic hepatitis C virus infection: A review

**DOI:** 10.1002/clc.23299

**Published:** 2019-11-30

**Authors:** Ahmed Babiker, Mohamed Hassan, Safwan Muhammed, Gregory Taylor, Bhawna Poonia, Anoop Shah, Shashwatee Bagchi

**Affiliations:** ^1^ Division of Infectious Diseases, Department of Medicine University of Pittsburgh Medical Center Pittsburgh Pennsylvania; ^2^ Division of General Internal Medicine, Department of Medicine University of Pittsburgh Medical Center Pittsburgh Pennsylvania; ^3^ Department of Medicine University of Maryland Medical Center Baltimore Maryland; ^4^ Institute of Human Virology University of Maryland School of Medicine Baltimore Maryland; ^5^ Department of Family Medicine University of Maryland School of Medicine Baltimore Maryland; ^6^ Division of Cardiology University of Edinburgh Little France Edinburgh; ^7^ Division of Infectious Diseases University of Maryland School of Medicine Baltimore Maryland

**Keywords:** biomarkers, cardiac biomarkers, chronic hepatitis C infection, hepatitis C, inflammatory biomarkers

## Abstract

Hepatitis C virus (HCV) infects 180 million people worldwide and over 4 million people in the United States. HCV infection is a major cause of chronic liver disease and is recognized as a risk factor for clinical cardiovascular disease (CVD). Many studies have shown increased prevalence of cardiac and inflammatory biomarkers in patients with chronic HCV infection (CHC), and though these markers may be used to risk stratify people for cardiac disease in the general population their role in the HCV population is unknown. Patients with CHC have elevated cardiac and inflammatory biomarkers compared to noninfected controls which may play a role in CVD risk stratification. We undertook a systematic review of inflammatory and cardiac biomarkers in people with HCV infection with a focus on the effect of CHC on serum levels of these markers and their utility as predictors of CVD in this population. Medline, EMBASE, and Cochrane databases were searched for relevant articles until June 2019. A total of 2430 results were reviewed with 115 studies included. Our review revealed that HCV infection significantly alters serum levels of markers of inflammation, endothelial function, and cardiac dysfunction prior to HCV treatment, and some of which may change in response to HCV therapy. Current risk stratification tools for development of CVD in the general population may not account for the increased inflammatory markers that appear to be elevated among HCV‐infected patients contributing to increased CVD risk.

**ABBREVIATIONS**
ALD: alcoholic liver diseases
ART: antiretroviral therapy
ASC HCV: asymptomatic HCV
BMI: body mass index
BNP: brain natriuretic peptide
CAD: coronary artery disease
CFR: coronary flow reserve
CHC: chronic hepatitis C
CHF: congestive Heart Failure
CLD: chronic liver disease
CRF: chronic renal failure
CRP: C‐reactive protein
cTn‐I: cardiac troponin I
cTn‐T: cardiac troponin T
CVA: cerebrovascular accident
CVD: cardiovascular disease
DAAs: directly acting antiviral agents
DM: diabetes mellitus
ESRD: end‐stage renal disease
GIB: gastrointestinal bleeding
gp: glycoprotein
HAI: hepatic activity index
HAV: hepatitis A virus
HBV: hepatitis B virus
HCV: hepatitis C virus
HD: hemodialysis
HDV: hepatitis D virus
HIV: human immunodeficiency virus
hsCRP: high‐sensitivity CRP
HTN: hypertension
IDU: injection drug use
IL: interleukin
INF‐ α: interferon alpha
LPS: lipoprotein polysaccharide
NR: nonresponders
NT‐proBNP: N‐terminal pro b‐type natriuretic peptide
OIs: opportunistic infections
sCD: soluble cellular differentiation
sE‐selectin: soluble E‐selectin
sICAM‐1: soluble intercellular adhesion molecule‐1
SLE: systemic lupus erythematosus
sP‐selectin: soluble P‐selectin
sTWEAK: soluble Tumor necrosis factor like weak inducer of apoptosis
sVCAM‐1: soluble vascular cell adhesion molecule‐1
SVR: sustained virologic response
TNF‐ alpha (or TNF‐α): tumor necrosis factor‐alpha
TNFR: TNF‐α receptors
TnI: troponin I
TnT: troponin T
TWEAK: TNF like weak inducer of apoptosis,
VL: viral load.

## 1 INTRODUCTION

Hepatitis C virus (HCV) is a single stranded RNA virus belonging to the Flaviviridae family. HCV has a global prevalence of 2.5% and infects 180 million people worldwide.^1^ In the United States, it is the most common blood borne infection affecting 0.8 persons in every 100 000 and causing significant morbidity and mortality.^2^ In 2016, over 2 million Americans had an opioid use disorder with 10% to 20% of those escalating to injection drug use.^3^ In this setting the prevalence of HCV has dramatically increased, especially in younger patients, with injection drug use (IDU) now being the primary mode of transmission in the US and a 2‐fold increase in the incidence rate of acute HCV infection.^4^

Chronic viral diseases, in particular human immunodeficiency virus (HIV), have been strongly linked to the development of clinical cardiac diseases.^5^, ^6^ Chronic HCV infection (CHC) has been linked to subclinical and clinical cardiovascular diseases (CVD), such as myocardial infarctions, congestive heart failure, cerebrovascular accident (CVA), and peripheral arterial disease,^7^ and proposed mechanisms include chronic inflammation and immune activation driven by HCV infection as well as direct endothelial invasion and dysfunction. Studies have shown increased prevalence of certain cardiac biomarkers associated with increased CVD risk in patients with CHC compared to age‐matched uninfected patients.^8^ Some of these biomarkers are combined with traditional risk factors to risk stratify persons for cardiac disease in the general population,^9^ but their utility in setting of HCV infection is unknown. Therefore, we aim to review the current literature on inflammatory and cardiac biomarkers in patients with HCV infection with a focus on the effect of CHC on serum levels of these markers and their utility as predictors of CVD in this population.

## 2 MATERIALS AND METHODS

We conducted a search based on PRISMA guidelines^10^ on Medline, EMBASE, and Cochrane for English language articles published through 14 June, 2019 using the following keywords and mesh terms: Hepatitis C, HCV, hepacivirus, chronic hepatitis C, inflammatory biomarkers, biomarkers and inflammation, biomarkers and inflammation mediators, cardiac biomarker, C‐reactive protein, CRP, high sensitivity C‐reactive protein, hsCRP, interluekin‐6, tumor necrosis factor‐alpha, troponin T, troponin I, brain natriuretic peptide, BNP, pro B‐type natriuretic peptide, N‐terminal pro b‐type natriuretic peptide, NT‐proBNP cardiovascular diseases, coronary disease, heart failure. All abstracts with the following inclusion criteria were reviewed: human studies in adults with CHC investigating serum levels of biomarkers of interest that included a HCV negative control group, and had full articles available for review. Study designs included randomized clinical trials, prospective, and retrospective observational cohorts, case‐control studies, cross‐sectional studies, and systemic reviews. Studies of acute hepatitis C and studies in children were excluded. Full‐length articles were reviewed by three independent reviewers (A.B., S.M., and M.H.), and any differences in reviewed data from articles were discussed and resolved by these reviewers and S.B. who reviewed these selected articles.

Cardiac biomarkers were categorized into three main groups: biomarkers of inflammation, biomarkers of endothelial function, and biomarkers of cardiac dysfunction.

## 3 RESULTS

The search performed in June 2019 yielded 1255 results on Medline, 1100 results on EMBASE, and 75 results on Cochrane giving a total of 2430 results. After duplicates were removed, 2394 references remained for review. Twenty‐four additional articles were added after performing ancestry and bibliography searches of all relevant articles, meta‐analyses, and systematic reviews. On review of titles and abstracts, 2156 were removed as they were found to be not relevant to our review. Of the remaining 262, 147 were removed because they did not meet eligibility criteria: 66 lacked a seronegative control group, 39 did not examine biomarkers of interest, 27 described only expression of and not serum levels of biomarkers, eight had relevant data missing, four were in a pediatric population, and three described levels of biomarkers following stimulation. Ultimately, 115 studies were included in our review (Figure S1).

### 3.1 Biomarkers of inflammation

Biomarkers of inflammation are commonly used to assess CVD risk in the general population. Fifty‐six studies evaluating the effect of HCV infection on inflammatory biomarkers were reviewed (Table 1 and Table S1) Biomarkers reviewed included interleukin‐6 (IL‐6), interleukin‐10 (IL‐10), tumor necrosis factor‐alpha (TNF‐α), TNF‐α receptors (TNFR), soluble CD163 (sCD163), and soluble CD14 (sCD14).

**Table 1:** Biomarkers of inflammation

Biomarker evaluated	Increased level in HCV vs controls		Similar levels in HCV vs controls	Decreased levels in HCV vs controls
IL‐6	Oliveira et al^31^ Falasca et al^32^ Helaly et al^33^ Khan et al^34^ Migita et al^36^ Zekri et al^37^ Malaguarnera et al^38^ Costantini et al^39^ Capone et al^40^ Oyanagi et al^41^ Lapinski et al^42^ Lecube et al^43^	Sandler et al^44^ ^a^ Grungreiff et al^46^ ^a^ Cotler et al^48^ ^a^ Hung et al^49^ Antonelli et al^50^ Shive et al^52^ Cua et al^53^ Grungreiff et al^54^ Lee et al^84^ Mishra et al^87^ Sousa et al^91^	Tsui et al^35^ Zuwała‐Jagiełło et al^57^ Mourtzikou et al^58^ Han et al^85^ Müller et al^92^	
IL‐6R	Migita et al^36^	Zekri et al^37^		
TNF‐α	Larrea et al^22^ ^a^ Oliveira *et al*.^31^ Falasca et al^32^ Helaly et al^33^ Khan et al^34^ Tsui et al^35^ Zekri et al^37^ Lecube et al^43^ Hung et al^49^ Cua et al^53^ Zuwała‐Jagiełło et al^57^ Mourtzikou et al^58^ Akcam et al^59^ Talaat et al^60^ Abdel‐Latif *et al* 61 Kallinowski et al^62^ ^a^ Al‐Jiffri^63^	Kaplanski et al^64^ Jia et al^65^ ^a^ El‐Bassiouni et al^66^ Antonelli et al^67^ Sayed‐Ahmed et al^69^ Toyoda et al^70^ ^a^ Kishihara et al^71^ ^a^ Raghuraman et al^72^ Valenti L et al^73^ Nelson et al^75^ Glowacki et al^76^ Riordan et al^77^ Zylberberg et al^78^ ^a^ Mishra et al^87^ Zografos et al^96^ ^a^ Zuwala‐Jagiello et al^103^	Cotler et al^48^ ^a^ Jia et al^65^ ^a^	
TNF‐RI/RII	Zekri et al^37^ Lecube et al^43^	Nelson et al^75^		
TNFR‐p55/p75	Kallinowski et al^62^ ^a^ Valenti et al^73^ Zylberberg et al^78^ ^a^ Itoh et al^79^ ^a^	Kakumu et al^80^ ^a^ Verma et al^81^ Farag et al^82^		
IL‐10	Falasca et al^32^ Khan et al^34^ Capone et al^40^ Hung et al^49^ Mourtzikou et al^58^ Akcam et al^59^ Jia et al^65^ ^a^	Kakumu et al^80^ ^a^ Verma et al^81^ Han et al^85^ Priimägi et al^86^ Mishra et al^87^ Fan et al^88^ Marín‐Serrano et al^89^ ^a^	Zekri et al^37^ Jia et al^65^ ^a^ Bruno et al^93^	Oliveira et al^31^
CRP/hsCRP	Roed et al^8^ Khan et al^34^ Zuwała‐Jagiełło et al^57^ Adinolfi et al^100^ Yilmaz et al^101^	Alyan et al^102^ Zuwala‐Jagiello et al^103^ Huang et al^104^ ^a^ Che et al^99^	Oguz et al^105^ ^a^	Tsui et al^35^ Moura et al^97^ Ufearo et al^98^
sCD14	Sandler et al^44^ ^a^	Markowtiz et al^83^	Farag et al^82^	
sCD163	Shive et al^52^			
Sgp130			Migita et al^36^	

a Assessed HCV therapy.

IL‐10 is an anti‐inflammatory cytokine that regulates the production of proinflammatory cytokines^11^ and down regulates the expression of adhesion molecules,^12^ and through these mechanisms may have anti‐atherosclerotic properties.^13^ The imbalance between anti‐ and pro‐inflammatory cytokines is thought to be critical in the pathogenesis of plaque formation and destabilization, though the results of clinical studies in angina patients remain inconclusive.^14^, ^15^ The same imbalance resulting in increased IL‐10 levels may be central to the persistence of HCV infection in CHC.^16^ IL‐6 is a proinflammatory cytokine that promotes activation and proliferation of lymphocytes and induction of hepatic acute phase proteins.^17^ Like IL‐10, IL‐6 has been linked to the development of atherosclerosis and serum levels have been correlated with cardiovascular disease and mortality.^18^

TNF‐α is a proinflammatory cytokine secreted by activated monocytes and macrophages in response to various infections. TNF‐α stimulates the release of acute phase proteins in the liver leading to lymphocyte and endothelial activation,^19^ and exerts its action through the binding of cellular TNF‐α receptor‐1 (TNFR‐1) and TNF‐R2.^20^ In healthy subjects, increased serum levels of TNF‐alpha have predicted CVD risk,^21^ and represented an independent risk factor for reduced event‐free survival.^21^ TNF‐dependent processes are up regulated and activated in CHC, and TNF‐α mRNA is ubiquitously expressed in hepatocytes, Kupffer cells, and infiltrating mononuclear cells at higher levels in CHC patients than in healthy controls.^22^, ^23^

C‐reactive protein (CRP) is an acute phase protein produced predominantly by hepatocytes, and influenced by IL‐6 and TNF‐α.^24^ Studies have shown significant association between increased CRP levels and underlying atherosclerosis, the risk of recurrent cardiovascular events among patients with established disease, and the incidence of first cardiovascular events among individuals at risk for atherosclerosis. CRP has been validated as one of the tools for assessment of CVD risk in the general population,^25^ and shown to correlate with survival and mortality in both non‐CHC^26^ and cirrhotic patients.^27^

Furthermore, monocyte/macrophage activation markers of immune activation such as soluble CD163 (sCD163)^28^ and soluble CD14 (sCD14)^29^ have been found to be associated with the burden of atherosclerosis and may predict mortality in HCV and HIV‐infected patients.^30^

Numerous studies have demonstrated increased expression of and serum levels of IL‐6,^31^, ^32^, ^33^, ^34^, ^35^, ^36^, ^37^, ^38^, ^39^, ^40^, ^41^, ^42^, ^43^, ^44^, ^45^, ^46^, ^47^, ^48^, ^49^, ^50^, ^51^, ^52^, ^53^, ^54^, ^55^, ^56^ TNF‐ α,^22^, ^31^, ^33^, ^35^, ^43^, ^45^, ^49^, ^53^, ^56^, ^57^, ^58^, ^59^, ^60^, ^61^, ^62^, ^63^, ^64^, ^65^, ^66^, ^67^, ^68^, ^69^, ^70^, ^71^, ^72^, ^73^, ^74^ sTNFR,^37^, ^43^, ^59^, ^61^, ^62^, ^72^, ^73^, ^75^, ^76^, ^77^, ^78^, ^79^, ^80^, ^81^, ^82^ sCD163,^52^ and sCD14^44^, ^52^, ^83^ among CHC patients compared to healthy controls (Table 1) and those with other hepatic diseases such as alcoholic liver disease.^84^ In addition, increased serum levels of IL‐10,^32^, ^34^, ^45^, ^47^, ^49^, ^56^, ^58^, ^59^, ^80^, ^81^, ^85^, ^86^, ^87^, ^88^, ^89^, ^90^ or disturbances in the ratios of proinflammatory/anti‐inflammatory cytokines TNF‐α/IL‐10 and IL‐6/IL‐10 have been noted among CHC patients compared to healthy controls.^31^ A few studies have demonstrated correlations between HCV viral load and cytokine levels.^34^, ^38^, ^80^, ^87^, ^91^

In a cohort of 981 patients with coronary heart disease enrolled in the Heart and Soul study, HCV seropositivity was associated with changes in levels of CRP, TNF‐α, IL‐6 increased Framingham risk scores,^31^ and hospitalizations due to clinical cardiac failure and death.^35^

However, other studies have reported different findings. These studies were smaller in size,^92^ and though unable to demonstrate differences between HCV groups and healthy controls they demonstrated increased serum levels of inflammatory markers with advanced HCV liver disease,^91^, ^93^ suggesting an evolution of inflammatory changes and cytokine imbalance in CHC as liver disease progresses.^94^ Other studies have also demonstrated increased hepatic expression and serum levels of IL‐10,^80^, ^81^, ^89^ IL‐6,^36^, ^37^, ^38^, ^39^, ^42^, ^91^, ^95^ TNF‐α,^23^, ^59^, ^60^, ^62^ and sTNFR^62^, ^79^, ^80^, ^81^ with increasing hepatic inflammation, steatosis, fibrosis, cirrhosis,^39^, ^78^, ^80^, ^96^ and the development of hepatocellular carcinoma (HCC).^40^

Studies have been mixed about the association between CRP levels and CHC infection. Some studies have demonstrated decreased CRP levels in CHC infection, which was postulated to be due to decreased production of CRP.^35^, ^97^, ^98^, ^99^ Ufearo et al found that CRP levels decreased as transferrin levels (also produced by hepatocytes) increased among HCV patients compared to controls, highlighting mechanisms other than poorly functioning hepatocytes.^98^

In contrast, other studies have shown elevated CRP levels in CHC infection.^8^, ^34^, ^57^, ^100^, ^101^, ^102^, ^103^ In a study of patients with angiographically documented CAD, CRP, and fibrinogen levels were significantly elevated in HCV‐infected patients compared to controls and HCV seropositivity was associated with increased severity of CAD.^103^ Similar results were reported with elevated high‐sensitivity CRP (hsCRP) levels and CVD in some studies,^103^, ^104^ whereas others found no differences in CRP levels among HCV patients and controls.^105^

Among the reported studies, few have linked altered levels of biomarkers with subclinical and clinical CVD in CHC. Tsui et al demonstrated increased levels of CRP, TNF‐α, IL‐6, and hospitalizations due to clinical cardiac failure among HCV positive patients compared to controls.^35^ Adinolfi et al demonstrated increased pro‐inflammatory cytokines levels were associated with a significantly higher prevalence of carotid atherosclerosis in HCV‐infected patients compared to controls.^100^ Similarly, Alyan et al found coronary artery disease (CAD) severity scores were significantly higher among CHC patients than among HCV negative controls. In the study, both HCV, CRP, and fibrinogen were significantly correlated to severity of CAD.^102^

Finally, many studies investigated the effect of HCV therapy on inflammatory markers and the majority demonstrated that HCV therapy significantly altered levels of inflammatory levels and/or predicted treatment success.^22^, ^46^, ^47^, ^48^, ^62^, ^65^, ^70^, ^71^, ^78^, ^79^, ^80^, ^89^, ^96^, ^105^

Taken the together, the preponderance of data shows a clear modulation of the immune system, an imbalance of pro‐and anti‐inflammatory biomarkers with a shift towards a proinflammatory state, and increased serum inflammatory levels among CHC‐infected patients. However, possibly due to blunted hepatocyte response and decreased production of CRP, CHC patients may score lower on CVD risk assessment models which rely heavily on CRP values.

### 3.2 Biomarkers of endothelial function

One of the early sentinel events in the development of atherosclerosis is endothelial dysfunction which results in an increased interaction between circulating leukocytes and the endothelium.^106^ This increased interaction and recruitment of circulating leukocytes is mediated by cellular adhesion molecules, and circulating forms of these molecules such as vascular cell adhesion molecule‐1 (VCAM‐1), endothelial‐leukocyte adhesion molecule‐1 (E‐selectin), intercellular adhesion molecule‐1 (ICAM‐1), and soluble selectin (sP‐selectin, sE‐selectin) have been found to predict endothelial dysfunction variably.^107^

Elevated levels of these markers have been correlated with increased risk of cardiovascular mortality in numerous studies in the general population.^8^, ^107^, ^108^, ^109^, ^110^, ^111^, ^112^, ^113^ In CHC patients, these adhesion molecules are expressed on sinusoidal cells and may be under the regulatory control of TNF‐alpha.^63^, ^114^

Six studies investigated the serum levels of markers of endothelial function among HCV patients (Table 2 and Table S2) Elevated levels of sICAM‐1,^8^, ^47^, ^63^, ^64^, ^66^ sVCAM‐1,^8^, ^63^, ^64^, ^66^, ^90^ sE‐selectin,^8^, ^63^ and sP‐selectin^47^ were uniformly increased across studies in CHC patients compared to controls.

**Table 2:** Biomarkers of endothelial function

Biomarker evaluated	Increased level in HCV vs controls	Similar levels in HCV vs controls	Decreased levels in HCV vs controls
sICAM‐1	Roed et al^8^ Panasiuk et al^47^ ^a^ Al‐Jiffri^63^ Kaplanski et al^64^ El‐Bassiouni et al^66^		
sVCAM‐1	Roed et al^8^ Al‐Jiffri^63^ Kaplanski et al^64^ El‐Bassiouni et al^66^ Micheloud et al^90^		
sE‐selectin	Roed et al^8^ Al‐Jiffri^63^ El‐Bassiouni et al^66^		
sP‐selectin	Panasiuk et al^47^ ^a^		

a Assessed HCV therapy.

In a study of 60 patients with CHC infection and age‐matched controls, higher levels of hsCRP, sICAM‐1, sVCAM‐1, and s‐E‐selectin were found among CHC patients compared to controls and increased biomarker levels correlated with carotid intima thickness.^8^ These markers of endothelial function have also been associated with liver disease progression, with higher levels associated with greater severity of liver disease,^44^, ^66^ and treatment response in CHC patients with levels decreasing after HCV therapy.^47^ More data is needed to determine whether these markers may be useful in the CVD risk assessment in the HCV patient population, as they are in the general population.

### 3.3 Biomarkers of cardiac dysfunction

Brain natriuretic peptide (BNP) and N‐terminal proBNP (NT‐proBNP) are natriuretic hormones that are primarily released by the ventricles of the heart. Plasma BNP provides prognostic information in patients with chronic heart failure and in those with asymptomatic or symptomatic left ventricular dysfunction.^115^, ^116^ Plasma NT‐proBNP has been shown to independently predict long‐term risk of death due to congestive heart failure.^117^, ^118^ Both markers have been established as reliable diagnostic and prognostic cardiac biomarkers that correlate with both symptoms of CHF and the severity of systolic and diastolic CHF in the general population.^119^, ^120^ In addition, these markers have been found to correlate with the degree of circulatory dysfunction in cirrhotic patients.^121^

Troponin T (TnT) and Troponin I (TnI) are cardiac proteins which regulate the calcium mediated interactions between actin and myosin^122^ released into the serum after myocardial injury. They have demonstrated prognostic value in a wide array of CVD, especially those associated with ischemic myocardial injury.^123^, ^124^

Nine studies evaluated serum levels of these biomarkers in CHC‐infected patients. (Table 3 and Table S3) Among CHC patients, elevated levels of BNP, NT‐proBNP,^50^, ^51^, ^67^, ^99^, ^125^, ^126^, ^127^, ^128^, ^129^ TnT, and TnI^103^, ^128^ have been observed compared to healthy controls.

**Table 3:** Biomarkers of cardiac dysfunction

Biomarker evaluated	Increased level in HCV vs controls	Similar levels in HCV vs controls	Decreased levels in HCV vs controls
NT‐proBNP	Antonelli et al^50^ Antonelli et al^67^ Che et al^99^ Antonelli et al^125^ Okada et al^126^ Wang et al^127^ Matsumori et al^128^ Che et al^129^		
cTn‐I/cTn‐T	Matsumori et al^128^		

Che et al found higher levels of NT‐ proBNP levels and a greater proportion of patients with impaired diastolic filling among HCV‐infected patients compared to controls, and the NT‐proBNP levels correlated with impaired diastolic filling.^129^ Okada et al found that CHC infection independently correlated with elevated levels of NT‐proBNP levels, and that patients with higher NT‐proBNP (>125 pg/mL) had significantly higher serum HCV RNA levels.^126^

The data above linking HCV infection with elevated biomarker levels and echocardiographic evidence of diastolic dysfunction suggest that HCV infection influences cardiac function asymptomatically and that testing with NT‐proBNP may have a role in identifying patients with low‐grade cardiac dysfunction and increased CVD mortality risk. There were no studies evaluating the change of these markers with HCV therapy.

## 4 SPECIAL POPULATIONS

### 4.1 HCV/HIV co‐infection

HIV infection has been strongly associated with increased risk of CVD through immune activation, exposure to antiretroviral therapy (ART) and disproportionately increased traditional risk factors for CVD among the HIV population.^130^, ^131^ HCV and HIV co‐infection have become increasingly common due to shared modes of transmission and become substantial in the current US opioid epidemic. HCV co‐infection among HIV patients has been shown to further increase the rate of CVD independent of other risk factors.^132^ Hence, monitoring CVD risk in the HIV/HCV co‐infected population may be especially important.

Sixteen studies have evaluated the effect of both HIV mono‐infection and HIV/HCV co‐infection on inflammatory biomarkers. (Table 4 and Table S4) The majority of studies have found that HCV co‐infection further increases the serum levels of IL‐6,^133^, ^134^, ^135^, ^136^, ^137^, ^138^ IL‐10,^139^ and sTNFRI^138^ but decreases levels of CRP or hsCRP^133^, ^134^, ^136^, ^140^, ^141^, ^142^ irrespective of liver function.^133^ Shah et al found that CRP levels fell with increasing IL‐6 levels suggesting attenuation of the CRP‐related IL‐6 response.^134^ Similar to HCV mono‐infected populations, these markers have been found to increase with progression of HCV liver disease^135^ and to correlate with mortality^143^ in co‐infected patients. However, there were no studies that evaluated the change in these inflammatory markers with HCV therapy among co‐infected patients. Forrester et al assessed lipid profiles and CRP levels in HCV mono‐infected, HIV/HCV co‐infected, and healthy controls and found no significant differences in CRP or lipid levels among the different groups.^144^ Their results conflicted with previous studies that found decreased lipid levels in co‐infected patients.^142^ No studies evaluated the change in these inflammatory markers with subclinical and/or clinical CVD among HIV/HCV co‐infected patients.

**Table 4:** Inflammatory and cardiovascular biomarkers in HCV/HIV co‐infection

Biomarker evaluated	Increase level in HCV vs controls	Similar levels in HCV vs controls	Decrease levels in HCV vs controls
IL‐6	Salter et al^133^ Shah et al^134^ Kohli et al^136^ Medrano et al^137^ de Oca Arjona et al^138^		
IL‐10	Garcia‐Broncano et al^139^		
TNF‐α	Garcia‐Broncano et al^139^ Dong et al^140^	Garcia‐Broncano et al^139^	
sTNFR‐1	Medrano et al^137^ Guzmán‐Fulgencio et al^147^ ^a^		
TNFR‐p55	de Oca Arjona et al^138^		
CRP/hsCRP	Dong et al^140^	Kohli et al^136^ Reingold et al^141^ Forrester et al^144^	Salter et al^133^ Shah et al^134^ Reingold et al^141^ Floris‐Morre et al^142^
sCD14	Medrano et al^137^ de Oca Arjona et al^138^ Shaked et al^148^		
sCD163	Beltran et al^149^ Mascia et al^150^		
sICAM‐1	Medrano et al^137^ De Castro et al^145^ ^a^ Masiá et al^146^ Guzmán‐Fulgencio et al^147^ ^a^		
sVCAM‐1	Medrano et al^137^ de Castro et al^145^ ^a^ Masiá et al^146^ de Larranaga et al^151^		
sE‐selectin	Guzmán‐Fulgencio et al^147^ ^a^		
sP‐selectin	de Larranaga et al^151^		
BNP	Dabrowska et al^152^		

a Assessed HCV therapy.

Studies of markers of endothelial function in HIV/HCV co‐infected patients have reported similar results to studies in HCV mono‐infected patients (Table 4 and Table S4) Studies have demonstrated elevated levels of sVCAM‐1,^137^, ^145^ sICAM‐1,^137^, ^145^, ^146^, ^147^ sCD14,^138^, ^148^ sCD163,^148^, ^149^, ^150^ sP‐selectin, and sE‐selectin^147^, ^151^ among co‐infected patients, which correlated with disease progression^145^ and treatment response,^145^ decreased with therapy,^145^, ^147^ predicted liver related mortality^143^ and were significantly associated with subclinical cardiovascular disease.^148^ Finally, one study reported significantly elevated BNP elevated levels among HIV/HCV co‐infected patients compared to HIV mono‐infected patients.^152^

Therefore, the totality of the data suggests that while HIV mono‐infection has a significant influence on serum cardiovascular biomarkers, HCV co‐infection may amplify this effect and further increase CVD risk. In addition, it may be possible to identify this increased risk in the HIV/HCV co‐infected population through measurement of select inflammatory and endothelial biomarkers.

### 4.2 CHC patients with other inflammatory comorbidities

The additive effect of comorbidities such as diabetes mellitus^153^ and end‐stage renal disease (ESRD) on the levels of inflammatory biomarkers may be important as well, since these markers may be elevated de novo in these specific patient populations.^154^ CHC infection is known to interfere with glucose and lipid metabolism, resulting in insulin resistance and DM^43^, ^155^ through interference of insulin signaling pathways related to increased TNF‐α^74^, ^156^ and higher HCV VL.^69^ HCV infection was associated with increased levels of TNF‐α and decreased levels of CRP in diabetic HCV‐infected patients compared to HCV uninfected diabetics, indicating modulation of HCV infection on the chronic inflammatory state in DM and underscoring the need to account for this co‐morbidity when assessing CVD through standard risk scoring.^68^, ^74^, ^157^ (Table 5 and Table S5).

**Table 5:** Inflammatory biomarkers in CHC patients with other inflammatory comorbidities

Biomarker evaluated	Increased level in HCV vs controls	Similar levels in HCV vs controls	Decreased levels in HCV vs controls
IL‐6	Pimentel et al^45^ Antonelli et al^51^ Caliskan et al^55^ Cielecka‐Kuszyk et al^56^ Falasca et al^164^ Siloşi et al^166^ Ramos‐Casals et al^167^ Riccio et al^169^	Nascimento et al^162^ Afzal et al^163^ el‐Din et al^165^	
IL‐10	Pimentel et al^45^ Cielecka‐Kuszyk et al^56^ Ramos‐Casals et al^167^		
TNF‐ α	Pimentel et al^45^ Caliskan et al^55^ Cielecka‐Kuszyk et al^56^ Mezher et al^68^ Elsammak et al^74^ Pawlak et al^160^ Siloşi et al^166^ Ramos‐Casals et al^167^ Antonelli et al^168^		
sTNFRI/II	el‐Din et al^165^ Realdon et al^170^		
CRP/hsCRP	Caliskan et al^55^ Yelken et al^158^ Chennu et al^159^ Pawlak et al^160^	Caliskan et al^161^ Nascimento et al^162^ Afzal et al^163^	Skowronski et al^157^
NT‐proBNP	Antonelli et al^51^		

TNF‐α and IL‐6 have been elevated in ESRD patients with and without HCV compared to healthy controls.^55^ In contrast, studies among ESRD patients evaluating CRP levels in the CHC population have shown mixed results, with studies demonstrating increased levels,^55^, ^158^, ^159^, ^160^ similar levels,^161^, ^162^, ^163^ and lower levels^163^ of serum hsCRP among ESRD patients with HCV infection compared to uninfected controls. In one study, authors found similar levels of serum hsCRP and IL‐6 levels in the HCV‐infected patients compared to the uninfected patients with mean IL‐6/hsCRP ratio significantly lower in the HCV positive group, leading the authors to hypothesize that the liver may have a blunted response to IL‐6 in the presence of HCV infection.^162^

The immune activation associated with CHC infection can lead to the development of immune related complications, which may further influence the levels of inflammatory markers associated with CVD. Increased levels of IL‐6, IL‐10, TNF‐α, and sTNFR in HCV‐infected patients with complications such as cryoglobulinemia, Sjogren syndrome, lymphoproliferative diseases, hemophilia, and HCV related arthritis have been reported compared to CHC‐infected patients without such complications or to healthy controls.^45^, ^51^, ^56^, ^164^, ^165^, ^166^, ^167^, ^168^, ^169^, ^170^

Therefore, special consideration may be needed when interpreting serum inflammatory markers in HCV populations who have DM, ESRD, and/or concomitant immune complications. In these populations with HCV infection and inflammatory conditions, there we no studies evaluating the change of these markers with HCV treatment, and none evaluated the association of these markers to subclinical and/or clinical CVD.

## 5 DISCUSSION

We found that the preponderance of data suggested that HCV infection significantly alters serum levels of markers of inflammation, endothelial function, and cardiac dysfunction prior to HCV treatment, and some of which may change in response to HCV therapy. The majority of studies demonstrate an imbalance of pro‐and anti‐inflammatory biomarkers with a shift towards a proinflammatory state among CHC‐infected patients.

Clinical presentations of CVD are preceded by an asymptomatic period, which can be long and insidious, but during this period biomarkers can be critical in identifying pathological developments that may lead to clinical cardiac events. Biomarkers are useful tools to risk stratify patients for development of disease and to monitor disease outcomes in response to therapies. Cardiac biomarkers used for these purposes in the general population may be useful in the HCV population too. Since the majority of studies consistently found increased levels of the pro‐inflammatory markers IL‐6 and TNF‐α and markers of endothelial function and cardiac dysfunction among HCV‐infected compared to uninfected patients, incorporating these biomarkers into risk stratification tools may improve the ability to discern an individual's risk for developing CVD among HCV‐infected patients who were determined to be at intermediate risk based on standard CVD assessment tools using traditional markers such as CRP only.^171^

Traditional risk stratification tools for CVD have been established and widely adopted in routine practice. Risk scoring tools such as the atherosclerotic CVD (ASCVD) risk score incorporate traditional risk factors such as smoking, hypertension and DM, and though these risk factors certainly contribute to an individual's CVD risk they do not fully account for the increased atherosclerosis and CVD events among certain high‐risk groups such as the HCV‐infected patient population.^25^ CVD risk assessment tools have been shown to perform sub optimally in HIV‐infected patients^172^ likely due to increased systemic inflammation, endothelial dysfunction, and metabolic derangements, which may disproportionately drive the atherosclerotic process in these patients and are not well accounted for in most tools currently. In addition, lipid levels, which are a component of many CVD risk stratification tools are known to be decreased in CHC patients,^173^ and therefore may underestimate CVD risk in these patients. Inflammation is recognized as a major component of atherosclerosis among both HCV‐infected and HCV uninfected patients,^100^, ^174^ and there is a growing concern that CVD risk assessment using current risk scoring tools may be suboptimal for patients with heightened inflammatory states leading to an underestimation of risk in these patients.^175^

CRP is an established cardiac biomarker for increased CVD risk and has been included in traditional risk assessment algorithms. Treatment strategies based on hsCRP levels have resulted in reduced CVD events,^176^ and hsCRP levels are used to re‐classify patients with intermediate ASCVD risk currently.^25^ However, CRP levels were decreased in HCV patients compared to uninfected patients in many^35^, ^98^, ^99^, ^157^, ^163^ but not all studies.^34^, ^102^, ^158^, ^159^, ^163^ Inconsistencies in results may be due to differences in patient populations, study designs and types of assay used. For example, CRP levels appear to be higher among HCV‐infected patients with ESRD who may have higher CRP levels at baseline. Reduced CRP levels may be due to reduced hepatic synthesis of CRP or direct inhibition during HCV replication regardless of liver function,^133^, ^136^, ^151^ and the use of CRP may underestimate the CVD risk in these patients.

Another advantage of some nontraditional biomarkers is their relationship to CHC disease stage,^36^, ^37^, ^38^, ^39^, ^42^, ^91^, ^95^ response to HCV treatment and achievement of SVR.^46^, ^47^, ^48^ Numerous studies have reported decreased CVD clinical endpoints and mortality with HCV therapy.^7^ There were conflicting results about the change of these biomarkers in response to HCV treatment, and these differing results may have been due to heterogeneous study designs, variable study populations, and different treatment regimens among studies.^47^, ^104^, ^105^, ^147^ Despite this variability, it was clear that eradication of HCV does have an effect on the HCV driven inflammatory and immune responses with a general trend towards return to normal levels of these markers. Nonetheless, there were a few studies that assessed the changes in inflammatory and cardiac markers with HCV therapy, and in these studies changes in biomarkers with HCV treatment was associated with CVD.^46^, ^47^, ^65^, ^79^, ^80^, ^89^, ^145^, ^147^ This finding has especially significant implications in the current era of directly acting antiviral agent (DAA) therapy, since large numbers of patients can be cured of HCV infection offering hope that HCV therapy could potentially mitigate CVD risk. The ability to refine CVD risk further using select cardiac biomarkers based on HCV stage of disease and to recalculate risk in response to DAAs and achievement of HCV cure would provide a powerful tool in selecting the highest‐risk patients in whom to target CVD preventive and management strategies in the HCV patient population.

Ours is one of the first comprehensive reviews of the literature reporting on the associations between HCV and a wide array of nontraditional potential CVD biomarkers such as endothelial markers. Strengths of our review included an extensive, systematic review of the topic and inclusion of various study designs, thereby allowing us to include many studies with pertinent, valuable findings. Furthermore, it is the first review to examine the possible additive influence of common comorbidities such as HIV co‐infection, DM, and ESRD on CHC and their effects on serum cardiac biomarkers. In addition, our review reported on the effect of antiviral therapy on these cardiac markers, which is of particular interest in the DAA era. Only one other similar systemic review by Osibogun et al included HIV/HCV co‐infected patients but it included 28 studies only.^177^ Finally, we chose only to investigate the association between CHC and cardiac biomarkers already known to correlate with cardiac disease and mortality in the general population. Limitations of the review included heterogeneous study designs, but this was purposeful because we felt that excluding too many relevant studies in the pursuit of a meta‐analysis would compromise the focus of reporting comprehensive data on a heterogeneous group of biomarkers. In addition, the studies included heterogeneous study populations, variable inclusion and exclusion criteria, and outcomes with differing definitions of similar endpoints. Also, there was inconsistent and incomplete capturing of traditional CVD risk factors among studies, which may have affected the associations found between HCV infection and the index cardiac biomarkers. In some studies, patients were on therapy (ART or HCV therapy) which may attenuate the expression of and serum levels of biomarkers investigated. Such differences among studies made it challenging to reconcile differences in results and limited our ability to make firm conclusions about the predictive value of these biomarkers. Importantly, only some studies reported on the association of the change in inflammatory and cardiac biomarkers with subclinical or clinical cardiovascular disease, thereby limiting our ability to conclude on the association of the change of these biomarkers with cardiac disease in the HCV population compared to the general population. However, the markers selected in this review have been associated with subclinical and clinical cardiovascular disease in the general population already, and CHC has been associated with subclinical and clinical cardiac disease in previous studies.^6^, ^7^ Furthermore, the scope of our review did not account for the possible contribution of genetic variations leading to genetic predisposition of different ethnic groups and different HCV genotypes to CVD outcomes. In particular polymorphisms in cytokine genes or promoter regions may affect expression of inflammatory mediators and hence may affect CVD risk.^178^ Studies investigating genetic associations with inflammatory markers have been inconclusive and were not considered in the scope of this review. Finally, non‐English studies were not reviewed which may have excluded some significant findings.

In conclusion, inflammatory pathways are fundamental to the pathogenesis of atherosclerosis and the development of cardiac events, and CHC infection has been shown to heighten the state of inflammation in these patients. Current risk stratification tools for development of CVD in the general population do not account for many of the inflammatory markers that appear to be elevated among HCV‐infected patients, and therefore there is concern that the CVD risk in these patients may be underestimated. Given the burden of HCV infection both nationally and internationally, there is an urgent need to evaluate additional cardiac biomarkers that may potentially identify or better discriminate CVD risk among HCV‐infected patients more accurately than those in the current standard risk assessment tools. Further prospective studies are needed to confirm the predictive value of these biomarkers of interest in this patient population.

## CONFLICT OF INTEREST

The authors declare no potential conflict of interests.

## Supporting information


**Appendix S1.** Supporting Information.

**FIGURE S1** Prisma flow diagram

## References

[1] Ghany MG , Strader DB , Thomas DL , Seeff LB . Diagnosis, management, and treatment of hepatitis C: an update. Hepatology. 2009;49(4):1335‐1374. 19330875 10.1002/hep.22759 PMC7477893

[2] Rosenberg ES , Rosenthal EM , Hall EW , et al. Prevalence of hepatitis C virus infection in US states and the District of Columbia, 2013 to 2016. JAMA Netw Open. 2018;1:e186371‐e. 10.1001/jamanetworkopen.2018.6371 PMC6324373 30646319

[3] Center for Behavioral Health Statistics and Quality . Key Substance Use and Mental Health Indicators in the United States: Results from the 2016 National Survey on Drug Use and Health. Rockville, MD: Center for Behavioral Health Statistics and Quality; 2017.

[4] Zibbell JE , Asher AK , Patel RC , et al. Increases in acute hepatitis C virus infection related to a growing opioid epidemic and associated injection drug use, United States, 2004 to 2014. Am J Public Health. 2018;108(2):175‐181. 29267061 10.2105/AJPH.2017.304132 PMC5846578

[5] Lee KK , Stelzle D , Bing R , et al. Global burden of atherosclerotic cardiovascular disease in people with hepatitis C virus infection: a systematic review, meta‐analysis, and modelling study. Lancet Gastroenterol Hepatol. 2019;4(10):794‐804. 31377134 10.1016/S2468-1253(19)30227-4 PMC6734111

[6] Shah ASV , Stelzle D , Lee KK , et al. Global burden of atherosclerotic cardiovascular disease in people living with HIV. Circulation. 2018;138(11):1100‐1112. 29967196 10.1161/CIRCULATIONAHA.117.033369 PMC6221183

[7] Babiker A , Jeudy J , Kligerman S , Khambaty M , Shah A , Bagchi S . Risk of cardiovascular disease due to chronic hepatitis C infection: a review. J Clin Transl Hepatol. 2017;5(4):343‐362. 29226101 10.14218/JCTH.2017.00021 PMC5719192

[8] Roed T , Kristoffersen US , Knudsen A , et al. Increased prevalence of coronary artery disease risk markers in patients with chronic hepatitis C – a cross‐sectional study. Vasc Health Risk Manag. 2014;10:55‐62. 24482574 10.2147/VHRM.S53557 PMC3905100

[9] Zethelius B , Berglund L , Sundström J , et al. Use of multiple biomarkers to improve the prediction of death from cardiovascular causes. N Engl J Med. 2008;358:2107‐2116. 18480203 10.1056/NEJMoa0707064

[10] Moher D , Liberati A , Tetzlaff J , Altman DG , The PG . Preferred reporting items for systematic reviews and meta‐analyses: the PRISMA statement. PLoS Med. 2009;6(7):e1000097. 10.1371/journal.pmed.1000097 PMC2707599 19621072

[11] de Vries J . Immunosuppressive and anti‐inflammatory properties of interleukin 10. Ann Med. 1995;27(5):537‐541. 8541028 10.3109/07853899509002465

[12] Song S , Ling‐Hu H , Roebuck KA , Rabbi MF , Donnelly RP , Finnegan A . Interleukin‐10 inhibits interferon‐gamma‐induced intercellular adhesion molecule‐1 gene transcription in human monocytes. Blood. 1997;89(12):4461‐4469. 9192770

[13] Von Der Thusen JH , Kuiper J , Fekkes ML , De Vos P , Van Berkel TJ , Biessen EA . Attenuation of atherogenesis by systemic and local adenovirus‐mediated gene transfer of interleukin‐10 in LDLr−/− mice. FASEB J. 2001;15(14):2730‐2732. 11687507 10.1096/fj.01-0483fje

[14] Waehre T , Halvorsen B , Damas JK , et al. Inflammatory imbalance between IL‐10 and TNFalpha in unstable angina potential plaque stabilizing effects of IL‐10. Eur J Clin Investig. 2002;32(11):803‐810. 12423320 10.1046/j.1365-2362.2002.01069.x

[15] Smith DA , Irving SD , Sheldon J , Cole D , Kaski JC . Serum levels of the antiinflammatory cytokine interleukin‐10 are decreased in patients with unstable angina. Circulation. 2001;104(7):746‐749. 11502695 10.1161/hc3201.094973

[16] Osburn WO , Levine JS , Chattergoon MA , Thomas DL , Cox AL . Anti‐inflammatory cytokines, pro‐fibrogenic chemokines and persistence of acute HCV infection. J Viral Hepat. 2013;20(6):404‐413. 10.1111/jvh.12052. 23647957 PMC3793396

[17] Akira S , Kishimoto T . IL‐6 and NF‐IL6 in acute‐phase response and viral infection. Immunol Rev. 1992;127:25‐50. 1380488 10.1111/j.1600-065x.1992.tb01407.x

[18] Ridker PM , Rifai N , Stampfer MJ , Hennekens CH . Plasma concentration of interleukin‐6 and the risk of future myocardial infarction among apparently healthy men. Circulation. 2000;101(15):1767‐1772. 10769275 10.1161/01.cir.101.15.1767

[19] Tracey KJ , Cerami A . Tumor necrosis factor: a pleiotropic cytokine and therapeutic target. Annu Rev Med. 1994;45:491‐503. 8198398 10.1146/annurev.med.45.1.491

[20] Bradley JR . TNF‐mediated inflammatory disease. J Pathol. 2008;214(2):149‐160. 18161752 10.1002/path.2287

[21] Nikolopoulou A , Tousoulis D , Antoniades C , et al. Common community infections and the risk for coronary artery disease and acute myocardial infarction: evidence for chronic over‐expression of tumor necrosis factor alpha and vascular cells adhesion molecule‐1. Int J Cardiol. 2008;130(2):246‐250. 18063147 10.1016/j.ijcard.2007.08.052

[22] Larrea E , Garcia N , Qian C , Civeira MP , Prieto J . Tumor necrosis factor alpha gene expression and the response to interferon in chronic hepatitis C. Hepatology. 1996;23(2):210‐217. 8591843 10.1002/hep.510230203 PMC7165845

[23] Kasprzak A , Seidel J , Spachacz R , et al. Intracellular expression of pro‐inflammatory cytokines (IL‐1alpha, TNF‐alpha, and IL‐6) in chronic hepatitis C. Rocz Akad Med Bialymst. 2004;49(Suppl 1):207‐209. 15638425

[24] Kushner I . The phenomenon of the acute phase response. Ann NY Acad Sci. 1982;389:39‐48. 7046585 10.1111/j.1749-6632.1982.tb22124.x

[25] Stone NJ , Robinson JG , Lichtenstein AH , et al. 2013 ACC/AHA guideline on the treatment of blood cholesterol to reduce atherosclerotic cardiovascular risk in adults: a report of the American College of Cardiology/American Heart Association task force on practice guidelines. Circulation. 2014;129(25 Suppl 2):S1‐S45. 24222016 10.1161/01.cir.0000437738.63853.7a

[26] Kaptoge S , Di Angelantonio E , Lowe G , et al. C‐reactive protein concentration and risk of coronary heart disease, stroke, and mortality: an individual participant meta‐analysis. Lancet. 2010;375(9709):132‐140. 20031199 10.1016/S0140-6736(09)61717-7 PMC3162187

[27] Mortensen C , Andersen O , Krag A , Bendtsen F , Moller S . High‐sensitivity C‐reactive protein levels predict survival and are related to haemodynamics in alcoholic cirrhosis. Eur J Gastroenterol Hepatol. 2012;24(6):619‐626. 22441510 10.1097/MEG.0b013e328351db6e

[28] Burdo TH , Lo J , Abbara S , et al. Soluble CD163, a novel marker of activated macrophages, is elevated and associated with noncalcified coronary plaque in HIV‐infected patients. J Infect Dis. 2011;204(8):1227‐1236. 21917896 10.1093/infdis/jir520 PMC3203384

[29] Kelesidis T , Kendall MA , Yang OO , Hodis HN , Currier JS . Biomarkers of microbial translocation and macrophage activation: association with progression of subclinical atherosclerosis in HIV‐1 infection. J Infect Dis. 2012;206(10):1558‐1567. 23066162 10.1093/infdis/jis545 PMC3475633

[30] Sandler NG , Wand H , Roque A , et al. Plasma levels of soluble CD14 independently predict mortality in HIV infection. J Infect Dis. 2011;203(6):780‐790. 21252259 10.1093/infdis/jiq118 PMC3071127

[31] Oliveira CP , Kappel CR , Siqueira ER , et al. Effects of hepatitis C virus on cardiovascular risk in infected patients: a comparative study. Int J Cardiol. 2013;164:221‐226. 21784542 10.1016/j.ijcard.2011.07.016

[32] Falasca K , Ucciferri C , Dalessandro M , et al. Cytokine patterns correlate with liver damage in patients with chronic hepatitis B and C. Ann Clin Lab Sci. 2006;36(2):144‐150. 16682509

[33] Helaly GF , El‐Afandy NM . Influence of HCV infection on insulin‐like growth factor 1 and proinflammatory cytokines: association with risk for growth hormone resistance development. Egypt J Immunol. 2009;16(2):115‐124. 22059359

[34] Khan S , Bhargava A , Pathak N , Maudar KK , Varshney S , Mishra PK . Circulating biomarkers and their possible role in pathogenesis of chronic hepatitis B and C viral infections. Indian J Clin Biochem. 2011;26(2):161‐168. 22468043 10.1007/s12291-010-0098-7 PMC3107408

[35] Tsui JI , Whooley MA , Monto A , Seal K , Tien PC , Shlipak M . Association of Hepatitis C virus seropositivity with inflammatory markers and heart failure in persons with coronary heart disease: data from the heart and soul study. J Card Fail. 2009;15(5):451‐456. 19477406 10.1016/j.cardfail.2008.12.003 PMC2782758

[36] Migita K , Abiru S , Maeda Y , et al. Serum levels of interleukin‐6 and its soluble receptors in patients with hepatitis C virus infection. Hum Immunol. 2006;67:27‐32. 16698422 10.1016/j.humimm.2006.02.025

[37] Zekri AR , Ashour MS , Hassan A , Alam El‐Din HM , El‐Shehaby AM , Abu‐Shady MA . Cytokine profile in Egyptian hepatitis C virus genotype‐4 in relation to liver disease progression. World J Gastroenterol. 2005;11(42):6624‐6630. 16425355 10.3748/wjg.v11.i42.6624 PMC4355755

[38] Malaguarnera M , Di Fazio I , Romeo MA , Restuccia S , Laurino A , Trovato BA . Elevation of interleukin 6 levels in patients with chronic hepatitis due to hepatitis C virus. J Gastroenterol. 1997;32(2):211‐215. 9085170 10.1007/BF02936370

[39] Costantini S , Capone F , Guerriero E , Maio P , Colonna G , Castello G . Serum cytokine levels as putative prognostic markers in the progression of chronic HCV hepatitis to cirrhosis. Eur Cytokine Netw. 2010;21(4):251‐256. 21081303 10.1684/ecn.2010.0214

[40] Capone F , Costantini S , Guerriero E , et al. Serum cytokine levels in patients with hepatocellular carcinoma. Eur Cytokine Netw. 2010;21:99‐104. 20478763 10.1684/ecn.2010.0192

[41] Oyanagi Y , Takahashi T , Matsui S , et al. Enhanced expression of interleukin‐6 in chronic hepatitis C. Liver. 1999;19(6):464‐472. 10661679 10.1111/j.1478-3231.1999.tb00078.x

[42] Lapinski TW . The levels of IL‐1beta, IL‐4 and IL‐6 in the serum and the liver tissue of chronic HCV‐infected patients. Arch Immunol Ther Exp. 2001;49(4):311‐316. 11726034

[43] Lecube A , Hernández C , Genescà J , Simó R . Proinflammatory cytokines, insulin resistance, and insulin secretion in chronic hepatitis C patients: a case‐control study. Diabetes Care. 2006;29(5):1096‐1101. 16644643 10.2337/diacare.2951096

[44] Sandler NG , Koh C , Roque A , et al. Host response to translocated microbial products predicts outcomes of patients with HBV or HCV infection. Gastroenterology. 2011;141(4):1220‐30.e3. 21726511 10.1053/j.gastro.2011.06.063 PMC3186837

[45] Pimentel JP , Chaves DG , Araújo AR , et al. Anti‐inflammatory/regulatory cytokine microenvironment mediated by IL‐4 and IL‐10 coordinates the immune response in hemophilia a patients infected chronically with hepatitis C virus. J Med Virol. 2013;85(6):1009‐1018. 23591975 10.1002/jmv.23554

[46] Grungreiff K , Reinhold D , Ansorge S . Serum concentrations of sIL‐2R, IL‐6, TGF‐beta1, neopterin, and zinc in chronic hepatitis C patients treated with interferon‐alpha. Cytokine. 1999;11(12):1076‐1080. 10623433 10.1006/cyto.1999.0504

[47] Panasiuk A , Prokopowicz D , Panasiuk B . Monocyte chemotactic protein‐1 and soluble adhesion molecules as possible prognostic markers of the efficacy of antiviral treatment in chronic hepatitis C. World J Gastroenterol. 2004;10(24):3639‐3642. 15534921 10.3748/wjg.v10.i24.3639 PMC4612007

[48] Cotler SJ , Reddy KR , McCone J , et al. An analysis of acute changes in interleukin‐6 levels after treatment of hepatitis C with consensus interferon. J Interf Cytokine Res. 2001;21(12):1011‐1019. 10.1089/107999001317205132 11798458

[49] Hung C‐H , Lee C‐M , Chen C‐H , et al. Association of inflammatory and anti‐inflammatory cytokines with insulin resistance in chronic hepatitis C. Liver Int. 2009;29(7):1086‐1093. 19302182 10.1111/j.1478-3231.2009.01991.x

[50] Antonelli A , Ferri C , Ferrari SM , et al. Hepatitis C is associated with high levels of circulating N‐terminal pro‐brain natriuretic peptide and Interleukin‐6. Eur J Inflamm. 2012;10(1):133‐138.

[51] Antonelli A , Ferri C , Ferrari SM , et al. High circulating levels of N‐terminal pro‐brain natriuretic peptide and interleukin 6 in patients with mixed cryoglobulinemia. J Med Virol. 2010;82(2):297‐303. 20029800 10.1002/jmv.21636

[52] Shive CL , Judge CJ , Clagett B , et al. Pre‐vaccine plasma levels of soluble inflammatory indices negatively predict responses to HAV, HBV, and tetanus vaccines in HCV and HIV infection. Vaccine. 2018;36(4):453‐460. 29254840 10.1016/j.vaccine.2017.12.018 PMC5767517

[53] Cua IH , Hui JM , Bandara P , et al. Insulin resistance and liver injury in hepatitis C is not associated with virus‐specific changes in adipocytokines. Hepatology. 2007;46(1):66‐73. 17596870 10.1002/hep.21703

[54] Grungreiff K , Hebell T , Gutensohn K , Reinhold A , Reinhold D . Plasma concentrations of zinc, copper, interleukin‐6 and interferon‐gamma, and plasma dipeptidyl peptidase IV activity in chronic hepatitis C. Mol Med Rep. 2009;2(1):63‐68. 21475791 10.3892/mmr_00000062

[55] Caliskan Y , Yelken B , Ozkok A , et al. Lower serum prohepcidin levels associated with lower iron and erythropoietin requirements in hemodialysis patients with chronic hepatitis C. BMC Nephrol. 2012;13:56. 22768976 10.1186/1471-2369-13-56 PMC3496582

[56] Cielecka‐Kuszyk J , Siennicka J , Jablonska J , et al. Is interleukin‐8 an additional to histopathological changes diagnostic marker in HCV‐infected patients with cryoglobulinemia? Hepatol Int. 2011;5(4):934‐940. 21484116 10.1007/s12072-011-9268-9

[57] Zuwala‐Jagiello J , Pazgan‐Simon M , Simon K , Warwas M . Advanced oxidation protein products and inflammatory markers in liver cirrhosis: a comparison between alcohol‐related and HCV‐related cirrhosis. Acta Biochim Pol. 2011;58(1):59‐65. 21403920

[58] Mourtzikou A , Alepaki M , Stamouli M , Pouliakis A , Skliris A , Karakitsos P . Evaluation of serum levels of IL‐6, TNF‐α, IL‐10, IL‐2 and IL‐4 in patients with chronic hepatitis. Inmunología. 2014;33(2):41‐50.

[59] Akcam FZ , Tigli A , Kaya O , Ciris M , Vural H . Cytokine levels and histopathology in chronic hepatitis B and chronic hepatitis C. J Interf Cytokine Res. 2012;32(12):570‐574. 10.1089/jir.2012.0048 23067363

[60] Talaat RM . Soluble angiogenesis factors in sera of Egyptian patients with hepatitis C virus infection: correlation with disease severity. Viral Immunol. 2010;23(2):151‐157. 20373995 10.1089/vim.2009.0089

[61] Abdel‐Latif MS . Plasma levels of matrix metalloproteinase (MMP)‐2, MMP‐9 and tumor necrosis factor‐α in chronic hepatitis C virus patients. Open Microbiol J. 2015;9:136‐140. 26464613 10.2174/1874285801509010136 PMC4598372

[62] Kallinowski B , Haseroth K , Marinos G , et al. Induction of tumour necrosis factor (TNF) receptor type p55 and p75 in patients with chronic hepatitis C virus (HCV) infection. Clin Exp Immunol. 1998;111(2):269‐277. 9486392 10.1046/j.1365-2249.1998.00469.x PMC1904907

[63] Al‐Jiffri O . Adhesive molecules and inflammatory markers among hepatitis C virus Saudi patients. Eur J Gen Med. 2017;14(4):89‐93.

[64] Kaplanski G , Farnarier C , Payan MJ , Bongrand P , Durand JM . Increased levels of soluble adhesion molecules in the serum of patients with hepatitis C. correlation with cytokine concentrations and liver inflammation and fibrosis. Dig Dis Sci. 1997;42(11):2277‐2284. 9398806 10.1023/a:1018818801824

[65] Jia HY , Du J , Zhu SH , et al. The roles of serum IL‐18, IL‐10, TNF‐alpha and sIL‐2R in patients with chronic hepatitis C. Hepatobiliary Pancreat Dis Int. 2002;1(3):378‐382. 14607710

[66] El‐Bassiouni NEI , Mahmoud OM , El Ahwani EG , Ibrahim RA , El Bassiouny AEI . Monocyte adhesion molecules expression in patients with chronic hepatitis C liver disease. Mediterranean J Hematol Infect Dis. 2013;5(1):e2013054. 10.4084/MJHID.2013.054 PMC3787663 24106604

[67] Antonelli A , Ferri C , Ferrari SM , et al. N‐terminal pro‐brain natriuretic peptide and tumor necrosis factor‐alpha both are increased in patients with hepatitis C. J Interf Cytokine Res. 2010;30(5):359‐363. 10.1089/jir.2009.0059 20187770

[68] Mezher MN , Jawad DHA . Evaluation of tumor necrosis factor‐ alpha in hepatitis C virus with diabetes mellitus and non diabetes mellitus patients. Res J Pharm Technol. 2017;10(10):3260‐3263.

[69] Sayed‐Ahmed L , Kotb N , El‐Serogy H , El‐Shazly S , Eid M . TNF‐alpha and CXCL‐10 correlation with insulin resistance in patients with chronic hepatitis C virus infection. Egypt J Immunol. 2010;17(1):101‐111. 22053613

[70] Toyoda M , Kakizaki S , Horiguchi N , et al. Role of serum soluble Fas/soluble Fas ligand and TNF‐alpha on response to interferon‐alpha therapy in chronic hepatitis C. Liver. 2000;20(4):305‐311. 10959809 10.1034/j.1600-0676.2000.020004305.x

[71] Kishihara Y , Hayashi J , Yoshimura E , Yamaji K , Nakashima K , Kashiwagi S . IL‐1 beta and TNF‐alpha produced by peripheral blood mononuclear cells before and during interferon therapy in patients with chronic hepatitis C. Dig Dis Sci. 1996;41(2):315‐321. 8601375 10.1007/BF02093821

[72] Raghuraman S , Abraham P , Daniel HD , Ramakrishna BS , Sridharan G . Characterization of soluble FAS, FAS ligand and tumour necrosis factor‐alpha in patients with chronic HCV infection. J Clin Virol. 2005;34(1):63‐70. 16087126 10.1016/j.jcv.2005.01.009

[73] Valenti L , Pulixi E , Fracanzani AL , et al. TNFalpha genotype affects TNFalpha release, insulin sensitivity and the severity of liver disease in HCV chronic hepatitis. J Hepatol. 2005;43(6):944‐950. 16143422 10.1016/j.jhep.2005.05.035

[74] Elsammak M , Refai W , Elsawaf A , Abdel‐Fattah I , Abd Elatti E , Ghazal A . Elevated serum tumor necrosis factor alpha and ferritin may contribute to the insulin resistance found in HCV positive Egyptian patients. Curr Med Res Opin. 2005;21(4):527‐534. 15899101 10.1185/030079905X38141

[75] Nelson DR , Lim HL , Marousis CG , et al. Activation of tumor necrosis factor‐alpha system in chronic hepatitis C virus infection. Dig Dis Sci. 1997;42(12):2487‐2494. 9440625 10.1023/a:1018804426724

[76] Głowacki MK , Cieśla A , Cibor D , et al. Selected apoptotic markers in serum of patients with chronic viral hepatitis C. Przegl Lek. 2014;71(7):369‐373. 25338331

[77] Riordan SM , Skinner NA , Kurtovic J , et al. Toll‐like receptor expression in chronic hepatitis C: correlation with pro‐inflammatory cytokine levels and liver injury. Inflamm Res. 2006;55(7):279‐285. 16955390 10.1007/s00011-006-0082-0

[78] Zylberberg H , Rimaniol AC , Pol S , et al. Soluble tumor necrosis factor receptors in chronic hepatitis C: a correlation with histological fibrosis and activity. J Hepatol. 1999;30(2):185‐191. 10068094 10.1016/s0168-8278(99)80060-9

[79] Itoh Y , Okanoue T , Ohnishi N , et al. Serum levels of soluble tumor necrosis factor receptors and effects of interferon therapy in patients with chronic hepatitis C virus infection. Am J Gastroenterol. 1999;94(5):1332‐1340. 10235215 10.1111/j.1572-0241.1999.01083.x

[80] Kakumu S , Okumura A , Ishikawa T , et al. Serum levels of IL‐10, IL‐15 and soluble tumour necrosis factor‐alpha (TNF‐alpha) receptors in type C chronic liver disease. Clin Exp Immunol. 1997;109(3):458‐463. 9328122 10.1046/j.1365-2249.1997.4861382.x PMC1904782

[81] Verma V , Chakravarti A , Kar P . Cytokine levels of TGF‐β, IL‐10, and sTNFαRII in type C chronic liver disease. Dig Dis Sci. 2008;53(8):2233‐2237. 18080762 10.1007/s10620-007-0130-9

[82] Farag NH , Rashed HA , Hassan M , et al. Hepatitis C infection, cognition, and inflammation in an Egyptian sample. J Med Virol. 2011;83(2):261‐266. 21181920 10.1002/jmv.21879

[83] Markowitz M , Deren S , Cleland C , et al. Chronic hepatitis C virus infection and the proinflammatory effects of injection drug use. J Infect Dis. 2016;214(9):1376‐1382. 27521361 10.1093/infdis/jiw373 PMC5079368

[84] Lee SH , Jeong S‐H , Park YS , et al. Serum prohepcidin levels in chronic hepatitis C, alcoholic liver disease, and nonalcoholic fatty liver disease. Korean J Hepatol. 2010;16(3):288‐294. 20924211 10.3350/kjhep.2010.16.3.288 PMC3304592

[85] Han ZQ , Huang T , Deng YZ , Zhu GZ . Expression profile and kinetics of cytokines and chemokines in patients with chronic hepatitis C. Int J Clin Exp Med. 2015;8(10):17995‐18003. 26770394 PMC4694294

[86] Priimägi L , Tefanova V , Tallo T , Schmidt E . The role of serum Th1 and Th2 cytokines in patients with chronic hepatitis B and hepatitis C virus infection. Acta Med Litunanica. 2005;12:28‐31.

[87] Mishra PK , Bhargava A , Vashistha P , Varshney S , Maudar KK . Mediators of the immune system and their possible role in pathogenesis of chronic hepatitis B and C viral infections. Asian Pacific J Mol Biol Biotechnol. 2010;18:341‐349.

[88] Fan XG , Liu WE , Li CZ , et al. Circulating Th1 and Th2 cytokines in patients with hepatitis C virus infection. Mediat Inflamm. 1998;7(4):295‐297. 10.1080/09629359890992 PMC1781851 9792341

[89] Marín‐Serrano E , Rodríguez‐Ramos C , Díaz F , Martín‐Herrera L , Girón‐González JA . Modulation of the anti‐inflammatory interleukin 10 and of proapoptotic IL‐18 in patients with chronic hepatitis C treated with interferon alpha and ribavirin. J Viral Hepat. 2006;13(4):230‐234. 16611188 10.1111/j.1365-2893.2005.00679.x

[90] Micheloud D , Salcedo M , Banares R , et al. Serum levels of fibrosis biomarkers measured early after liver transplantation are associated with severe hepatitis C virus recurrence. Transplant Infect Dis. 2009;11(2):183‐188. 10.1111/j.1399-3062.2009.00370.x 19254326

[91] Sousa GM , Oliveira IS , Andrade LJ , Sousa‐Atta ML , Parana R , Atta AM . Serum levels of Th17 associated cytokines in chronic hepatitis C virus infection. Cytokine. 2012;60(1):138‐142. 22748467 10.1016/j.cyto.2012.06.003

[92] Müller C , Zielinski CC . Interleukin‐6 production by peripheral blood monocytes in patients with chronic liver disease and acute viral hepatitis. J Hepatol. 1992;15(3):372‐377. 1447505 10.1016/0168-8278(92)90071-v

[93] Bruno CM , Valenti M , Bertino G , et al. Relationship between circulating interleukin‐10 and histological features in patients with chronic C hepatitis. Ann Saudi Med. 2011;31(4):360‐364. 21808111 10.4103/0256-4947.83215 PMC3156511

[94] McCaughan GW , George J . Fibrosis progression in chronic hepatitis C virus infection. Gut. 2004;53(3):318‐321. 14960506 10.1136/gut.2003.026393 PMC1773949

[95] Oyanagi Y , Takahashi T , Matsui S , et al. Ehanced expression of interleukin‐6 in chronic hepatitis C. Liver. 1992;19:464‐472. 10.1111/j.1478-3231.1999.tb00078.x 10661679

[96] Zografos TA , Liaskos C , Rigopoulou EI , et al. Adiponectin: a new independent predictor of liver steatosis and response to IFN‐alpha treatment in chronic hepatitis C. Am J Gastroenterol. 2008;103(3):605‐614. 18190648 10.1111/j.1572-0241.2007.01729.x

[97] Moura TCF , Amoras E , Queiroz MAF , et al. Association of serum levels of C‐reactive protein with CRP‐717 T/C polymorphism and viremia in HCV and HBV carriers. Rev Soc Bras Med Trop. 2019;52:e20180455. 10.1590/0037-8682-0455-2018 30810658

[98] Ufearo H , Kambal K , Onojobi GO , et al. Complete blood count, measures of iron status and inflammatory markers in inner‐city African Americans with undiagnosed hepatitis C seropositivity. Clin Chim Acta. 2010;411(9–10):653‐656. 20117104 10.1016/j.cca.2010.01.028 PMC2847047

[99] Che W , Zhang B , Liu W , Wei Y , Xu Y , Hu D . Association between high‐sensitivity C‐reactive protein and N‐terminal pro‐B‐type natriuretic peptide in patients with hepatitis C virus infection. Mediat Inflamm. 2012;2012:730923. 10.1155/2012/730923 PMC3356947 22645408

[100] Adinolfi LE , Restivo L , Guerrera B , et al. Chronic HCV infection is a risk factor of ischemic stroke. Atherosclerosis. 2013;231(1):22‐26. 24125405 10.1016/j.atherosclerosis.2013.08.003

[101] Yilmaz S , Bayan K , Tuzun Y , et al. Replacement of hystological findings: serum hyaluronic acid for fibrosis, high‐sensitive C‐reactive protein for necroinflamation in chronic viral hepatitis. Int J Clin Pract. 2007;61(3):438‐443. 17313611 10.1111/j.1742-1241.2006.00912.x

[102] Alyan O , Kacmaz F , Ozdemir O , et al. Hepatitis C infection associated with increased coronary artery atherosclerosis defined by modified Reardson severity score system. Circulation. 2008;72(12):1960‐1965. 10.1253/circj.cj-08-0459 18957787

[103] Zuwala‐Jagiello J , Murawska‐Cialowicz E , Pazgan‐Simon M . Increased circulating advanced oxidation protein products and high‐sensitive troponin T in cirrhotic patients with chronic hepatitis C: a preliminary report. Biomed Res Int. 2015;2015:786570. 26665009 10.1155/2015/786570 PMC4668303

[104] Huang CF , Hsieh MY , Yang JF , et al. Serum hs‐CRP was correlated with treatment response to pegylated interferon and ribavirin combination therapy in chronic hepatitis C patients. Hepatol Int. 2010;4(3):621‐627. 21063486 10.1007/s12072-010-9200-8 PMC2939996

[105] Oguz A , Atay AE , Tas A , Seven G , Koruk M . Predictive role of acute phase reactants in the response to therapy in patients with chronic hepatitis C virus infection. Gut Liver. 2013;7(1):82‐88. 23424009 10.5009/gnl.2013.7.1.82 PMC3572325

[106] Borissoff JI , Spronk HMH , Heeneman S , Ten Cate H . Is thrombin a key player in the “coagulation‐atherogenesis” maze? Cardiovasc Res. 2009;82:392‐403. 19228706 10.1093/cvr/cvp066

[107] Hwang SJ , Ballantyne CM , Sharrett AR , et al. Circulating adhesion molecules VCAM‐1, ICAM‐1, and E‐selectin in carotid atherosclerosis and incident coronary heart disease cases: the atherosclerosis risk in communities (ARIC) study. Circulation. 1997;96(12):4219‐4225. 9416885 10.1161/01.cir.96.12.4219

[108] Toss H , Lindahl B , Siegbahn A , Wallentin L . Prognostic influence of increased fibrinogen and C‐reactive protein levels in unstable coronary artery disease. FRISC study group. Fragmin during instability in coronary artery disease. Circulation. 1997;96(12):4204‐4210. 9416883 10.1161/01.cir.96.12.4204

[109] O'Malley T , Ludlam CA , Riemermsa RA , Fox KA . Early increase in levels of soluble inter‐cellular adhesion molecule‐1 (sICAM‐1); potential risk factor for the acute coronary syndromes. Eur Heart J. 2001;22(14):1226‐1234. 11440495 10.1053/euhj.2000.2480

[110] Ridker PM , Hennekens CH , Buring JE , Rifai N . C‐reactive protein and other markers of inflammation in the prediction of cardiovascular disease in women. N Engl J Med. 2000;342(12):836‐843. 10733371 10.1056/NEJM200003233421202

[111] Ridker PM , Hennekens CH , Roitman‐Johnson B , Stampfer MJ , Allen J . Plasma concentration of soluble intercellular adhesion molecule 1 and risks of future myocardial infarction in apparently healthy men. Lancet. 1998;351(9096):88‐92. 9439492 10.1016/S0140-6736(97)09032-6

[112] Blankenberg S , Rupprecht HJ , Bickel C , et al. Circulating cell adhesion molecules and death in patients with coronary artery disease. Circulation. 2001;104(12):1336‐1342. 11560847 10.1161/hc3701.095949

[113] Schmidt C , Hulthe J , Fagerberg B . Baseline ICAM‐1 and VCAM‐1 are increased in initially healthy middle‐aged men who develop cardiovascular disease during 6.6 years of follow‐up. Angiology. 2009;60(1):108‐114. 18504269 10.1177/0003319708316899

[114] Simpson KJ , Hayes PC . Soluble adhesion molecules in immune mediated liver disease. Gut. 1995;36(6):806‐808. 7615263 10.1136/gut.36.6.806 PMC1382612

[115] Anand IS , Fisher LD , Chiang YT , et al. Changes in brain natriuretic peptide and norepinephrine over time and mortality and morbidity in the valsartan heart failure trial (Val‐HeFT). Circulation. 2003;107:1278‐1283. 12628948 10.1161/01.cir.0000054164.99881.00

[116] Tsutamoto T , Wada A , Maeda K , et al. Plasma brain natriuretic peptide level as a biochemical marker of morbidity and mortality in patients with asymptomatic or minimally symptomatic left ventricular dysfunction. Comparison with plasma angiotensin II and endothelin‐1. Eur Heart J. 1999;20(24):1799‐1807. 10581138 10.1053/euhj.1999.1746

[117] Chen AA , Wood MJ , Krauser DG , et al. NT‐proBNP levels, echocardiographic findings, and outcomes in breathless patients: results from the ProBNP investigation of dyspnoea in the emergency department (PRIDE) echocardiographic substudy. Eur Heart J. 2006;27(7):839‐845. 16510467 10.1093/eurheartj/ehi811

[118] Moe GW , Howlett J , Januzzi JL , Zowall H . N‐terminal pro‐B‐type natriuretic peptide testing improves the management of patients with suspected acute heart failure: primary results of the Canadian prospective randomized multicenter IMPROVE‐CHF study. Circulation. 2007;115(24):3103‐3110. 17548729 10.1161/CIRCULATIONAHA.106.666255

[119] Doust JA , Glasziou PP , Pietrzak E , Dobson AJ . A systemic review of the diagnostic accuracy of naturetic peptides for heart failure. Arch Intern Med. 2004;164(18):1978‐1984. 15477431 10.1001/archinte.164.18.1978

[120] Clerico A , Emdin M . Diagnostic accuracy and prognostic relevance of the measurement of cardiac naturetic peptides: a review. Clin Chem. 2004;50(1):33‐50. 14633912 10.1373/clinchem.2003.024760

[121] Henriksen JH , Gotze JP , Fuglsang S , Christensen E , Bendtsen F , Moller S . Increased circulating pro‐brain natriuretic peptide (proBNP) and brain natriuretic peptide (BNP) in patients with cirrhosis: relation to cardiovascular dysfunction and severity of disease. Gut. 2003;52(10):1511‐1517. 12970147 10.1136/gut.52.10.1511 PMC1773816

[122] Sharma S , Jackson PG , Makan J . Cardiac troponins. J Clin Pathol. 2004;57(10):1025‐1026. 15452153 10.1136/jcp.2003.015420 PMC1770452

[123] Sato Y , Yamada T , Taniguchi R , et al. Persistently increased serum concentrations of cardiac troponin t in patients with idiopathic dilated cardiomyopathy are predictive of adverse outcomes. Circulation. 2001;103(3):369‐374. 11157687 10.1161/01.cir.103.3.369

[124] Wiese S , Mortensen C , Gøtze JP , et al. Cardiac and proinflammatory markers predict prognosis in cirrhosis. Liver Int. 2014;34(6):e19‐e30. 10.1111/liv.12428 24313898

[125] Antonelli A , Ferri C , Ferrari SM , et al. High levels of circulating N‐terminal pro‐brain natriuretic peptide in patients with hepatitis C. J Viral Hepat. 2010;17(12):851‐853. 20002300 10.1111/j.1365-2893.2009.01237.x

[126] Okada K , Furusyo N , Ogawa E , et al. Association between chronic hepatitis C virus infection and high levels of circulating N‐terminal pro‐brain natriuretic peptide. Endocrine. 2013;43(1):200‐205. 22581254 10.1007/s12020-012-9688-x

[127] Wang L , Geng J , Li J , et al. The biomarker N‐terminal pro‐brain natriuretic peptide and liver diseases. Clin Invest Med. 2011;34(1):E30‐E37. 21291633 10.25011/cim.v34i1.14910

[128] Matsumori A , Shimada T , Chapman NM , Tracy SM , Mason JW . Myocarditis and heart failure associated with hepatitis C virus infection. J Card Fail. 2006;12(4):293‐298. 16679263 10.1016/j.cardfail.2005.11.004

[129] Che W , Liu W , Wei Y , et al. Increased serum N‐terminal pro‐B‐type natriuretic peptide and left ventricle diastolic dysfunction in patients with hepatitis C virus infection. J Viral Hepat. 2012;19(5):327‐331. 22497811 10.1111/j.1365-2893.2011.01551.x

[130] Hickey A , Bagchi S . Cardiovascular disease risk assessment tools in HIV‐infected patients ‐ are they adequate? J AIDS Clin Res. 2016;2016:7‐583.

[131] Burrowes S , Cahill P , Kottilil S , Bagchi S . Contribution of antiretroviral therapy to cardiovascular disease risk in HIV‐infected patients. Futur Virol. 2016;11(7):509‐527.

[132] Bagchi S , Burrowes SA , Fantry LE , et al. Factors associated with high cardiovascular risk in a primarily African American, urban HIV‐infected population. SAGE Open Med. 2017;5:2050312117725644. 28839941 10.1177/2050312117725644 PMC5557160

[133] Salter ML , Lau B , Mehta SH , Go VF , Leng S , Kirk GD . Correlates of elevated interleukin‐6 and C‐reactive protein in persons with or at high risk for HCV and HIV infections. J Acquir Immune Defic Syndr. 2013;64(5):488‐495. 23978997 10.1097/QAI.0b013e3182a7ee2e PMC3848037

[134] Shah S , Ma Y , Scherzer R , et al. Association of HIV, hepatitis C virus and liver fibrosis severity with interleukin‐6 and C‐reactive protein levels. AIDS. 2015;29(11):1325‐1333. 25870985 10.1097/QAD.0000000000000654 PMC4478137

[135] Peters L , Neuhaus J , Duprez D , et al. Biomarkers of inflammation, coagulation and microbial translocation in HIV/HCV co‐infected patients in the SMART study. J Clin Virol. 2014;60(3):295‐300. 24793968 10.1016/j.jcv.2014.03.017

[136] Kohli P , Ganz P , Ma Y , et al. HIV and hepatitis C‐Coinfected patients have lower low‐density lipoprotein cholesterol despite higher Proprotein Convertase Subtilisin Kexin 9 (PCSK9): an apparent "PCSK9‐lipid paradox". J Am Heart Assoc. 2016;5(5):pii: e002683. 10.1161/JAHA.115.002683 PMC4889164 27130349

[137] Medrano LM , Garcia‐Broncano P , Berenguer J , et al. Elevated liver stiffness is linked to increased biomarkers of inflammation and immune activation in HIV/hepatitis C virus‐coinfected patients. AIDS. 2018;32(9):1095‐1105. 29438197 10.1097/QAD.0000000000001787

[138] de Oca Arjona MM , Marquez M , Soto MJ , et al. Bacterial translocation in HIV‐infected patients with HCV cirrhosis: implication in hemodynamic alterations and mortality. J Acquir Immune Defic Syndr. 2011;56(5):420‐427. 21266909 10.1097/QAI.0b013e31820ef408

[139] Garcia‐Broncano P , Medrano LM , Berenguer J , et al. Dysregulation of the immune system in HIV/HCV‐Coinfected patients according to liver stiffness status. Cell. 2018;7(11):196. 10.3390/cells7110196 PMC6262386 30400258

[140] Dong Y , Zhi X , Lei G . Changes of body immunity and inflammatory response in HIV/HCV co‐infected patients. Exp Ther Med. 2019;17(1):403‐407. 30651812 10.3892/etm.2018.6938 PMC6307362

[141] Reingold J , Wanke C , Kotler D , et al. Association of HIV infection and HIV/HCV coinfection with C‐reactive protein levels: the fat redistribution and metabolic change in HIV infection (FRAM) study. J Acquir Immune Defic Syndr. 2008;48(2):142‐148. 18344877 10.1097/QAI.0b013e3181685727 PMC2561207

[142] Floris‐Moore M , Howard AA , Lo Y , Schoenbaum EE , Arnsten JH , Klein RS . Hepatitis C infection is associated with lower lipids and high‐sensitivity C‐reactive protein in HIV‐infected men. AIDS Patient Care STDs. 2007;21(7):479‐491. 17651029 10.1089/apc.2006.0150 PMC2423809

[143] Nunes D , Fleming C , Offner G , et al. Noninvasive markers of liver fibrosis are highly predictive of liver‐related death in a cohort of HCV‐infected individuals with and without HIV infection. Am J Gastroenterol. 2010;105(6):1346‐1353. 20179698 10.1038/ajg.2009.746

[144] Forrester J , McGovern B , Rhee M , Sterling R . The individual and combined influence of HIV and hepatitis C virus on dyslipdemia in a high risk popuation. HIV Med. 2009;10(9):555‐563. 19496835 10.1111/j.1468-1293.2009.00722.x PMC2782864

[145] de Castro IF , Micheloud D , Berenguer J , et al. Hepatitis C virus infection is associated with endothelial dysfunction in HIV/hepatitis C virus coinfected patients. AIDS. 2010;24(13):2059‐2067. 20616694 10.1097/QAD.0b013e32833ce54d

[146] Masiá M , Padilla S , Robledano C , Ramos JM , Gutiérrez F . Evaluation of endothelial function and subclinical atherosclerosis in association with hepatitis C virus in HIV‐infected patients: a cross‐sectional study. BMC Infect Dis. 2011;11(1):265. 21967471 10.1186/1471-2334-11-265 PMC3198698

[147] Guzmán‐Fulgencio M , Berenguer J , Fernandez de Castro I , et al. Sustained virological response to interferon‐α plus ribavirin decreases inflammation and endothelial dysfunction markers in HIV/HCV co‐infected patients. J Antimicrob Chemother. 2011;66(3):645‐649. 21393232 10.1093/jac/dkq518

[148] Shaked I , Hanna DB , Gleissner C , et al. Macrophage inflammatory markers are associated with subclinical carotid artery disease in women with human immunodeficiency virus or hepatitis C virus infection. Arterioscler Thromb Vasc Biol. 2014;34(5):1085‐1092. 24651679 10.1161/ATVBAHA.113.303153 PMC4067091

[149] Beltran LM , Munoz Hernandez R , de Pablo Bernal RS , et al. Reduced sTWEAK and increased sCD163 levels in HIV‐infected patients: modulation by antiretroviral treatment, HIV replication and HCV co‐infection. PLoS One. 2014;9(3):e90541. 10.1371/journal.pone.0090541 PMC3942443 24594990

[150] Mascia C , Lichtner M , Zuccala P , et al. Active HCV infection is associated with increased circulating levels of interferon‐gamma (IFN‐gamma)‐inducible protein‐10 (IP‐10), soluble CD163 and inflammatory monocytes regardless of liver fibrosis and HIV coinfection. Clin Res Hepatol Gastroenterol. 2017;41(6):644‐655. 28578937 10.1016/j.clinre.2017.04.007

[151] de Larranaga GF , Wingeyer SD , Puga LM , Alonso BS , Benetucci JA . Relationship between hepatitis C virus (HCV) and insulin resistance, endothelial perturbation, and platelet activation in HIV‐HCV‐coinfected patients under highly active antiretroviral treatment. Eur J Clin Microbiol Infect Dis. 2006;25(2):98‐103. 16477441 10.1007/s10096-006-0090-6

[152] Dabrowska MM , Mikula T , Wiercinska‐Drapalo A . HCV coinfection possibly promotes left ventricular dysfunction development: analysis of brain natriuretic peptide serum levels in HCV/HIV‐coinfected and HIV‐monoinfected patients. Eur J Gastroenterol Hepatol. 2012;24(11):1308‐1312. 22825646 10.1097/MEG.0b013e32835702c6

[153] Feldmann G , Nischalke HD , Nattermann J , et al. Induction of interleukin‐6 by hepatitis C virus core protein in hepatitis C‐associated mixed cryoglobulinemia and B‐cell non‐Hodgkin's lymphoma. Clin Cancer Res. 2006;12(15):4491‐4498. 16899594 10.1158/1078-0432.CCR-06-0154

[154] Kilpatrick ES , Keevil BG , Jagger C , Spooner RJ , Small M . Determinants of raised C‐reactive protein concentration in type 1 diabetes. QJM. 2000;93(4):231‐236. 10787451 10.1093/qjmed/93.4.231

[155] Negro F . Facts and fictions of HCV and comorbidities: steatosis, diabetes mellitus, and cardiovascular diseases. J Hepatol. 2014;61(1):69‐78. 10.1016/j.jhep.2014.08.003 25443347

[156] Noto H , Raskin P . Hepatitis C infection and diabetes. J Diabetes Complicat. 2006;20(2):113‐120. 16504840 10.1016/j.jdiacomp.2006.01.001

[157] Skowronski M , Zozulinska‐Ziolkiewicz D , Juszczyk J , Michalski W , Wierusz‐Wysocka B . C‐reactive protein and chronic hepatitis C virus infection in diabetic patients. Pol Arch Med Wewn. 2010;120(12):491‐496. 21178905

[158] Yelken B , Gorgulu N , Caliskan Y , et al. Association between chronic hepatitis C infection and coronary flow reserve in dialysis patients with failed renal allografts. Transplant Proc. 2009;41(5):1519‐1523. 19545669 10.1016/j.transproceed.2009.03.069

[159] Chennu KK , Devi NH , Rao PS , Lakshmi A , Kumar VS . Markers of oxidative stress, inflammation and endothelial dysfunction in ESRD patients with hepatitis‐C virus positive on maintenance haemodialysis. J Clin Diagn Res. 2018;12(6):17‐20.

[160] Pawlak K , Mysliwiec M , Pawlak D . Chronic viral hepatitis and iron affect the plasma levels of LIGHT–a new member of the TNF superfamily in uraemic haemodialyzed patients. Cytokine. 2007;39(3):201‐206. 17827030 10.1016/j.cyto.2007.07.189

[161] Caliskan Y , Oflaz H , Pusuroglu H , et al. Hepatitis C virus infection in hemodialysis patients is not associated with insulin resistance, inflammation and atherosclerosis. Clin Nephrol. 2009;71(2):147‐157. 19203507 10.5414/cnp71147

[162] Nascimento MM , Bruchfeld A , Suliman ME , et al. Effect of hepatitis C serology on C‐reactive protein in a cohort of Brazilian hemodialysis patients. Braz J Med Biol Res. 2005;38:783‐788. 15917961 10.1590/s0100-879x2005000500017

[163] Afzal N , Abbas S , Ahmed A , Arif M , Javeed K . Effect of hepatitis C virus on C‐reactive protein and interleukin‐6 in hemodialysis patients. Iran J Kidney Dis. 2011;5(3):182‐186. 21525578

[164] Falasca K , Mancino P , Ucciferri C , et al. Inflammatory cytokines and S‐100b protein in patients with hepatitis C infection and cryoglobulinemias. Clin Invest Med. 2007;30(5):E167‐E176. 17892758 10.25011/cim.v30i5.2892

[165] El‐Din HM , Attia MA , Hamza MR , Khaled HM , Thoraya MA , Eisa SA . Hepatitis C virus and related changes in immunological parameters in non Hodgkin's lymphoma patients. Egypt J Immunol. 2004;11(1):55‐64. 15724387

[166] Siloşi I , Boldeanu L , Biciuşcă V , et al. Serum biomarkers for discrimination between hepatitis C‐related Arthropathy and early rheumatoid arthritis. Int J Mol Sci. 2017;18(6):1304. 10.3390/ijms18061304 PMC5486125 28629188

[167] Ramos‐Casals M , Garcia‐Carrasco M , Cervera R , et al. Th1/Th2 cytokine imbalance in patients with Sjogren syndrome secondary to hepatitis C virus infection. Semin Arthritis Rheum. 2002;32(1):56‐63. 12219321 10.1053/sarh.2002.33724

[168] Antonelli A , Ferri C , Fallahi P , et al. High values of CXCL10 serum levels in mixed cryoglobulinemia associated with hepatitis C infection. Am J Gastroenterol. 2008;103(10):2488‐2494. 18775023 10.1111/j.1572-0241.2008.02040.x

[169] Riccio A , Postiglione L , Sabatini P , et al. Similar serum levels of IL‐6 and its soluble receptors in patients with HCV‐related arthritis and rheumatoid arthritis: a pilot study. Int J Immunopathol Pharmacol. 2012;25(1):281‐285. 22507342 10.1177/039463201202500132

[170] Realdon S , Pontisso P , Adami F , et al. High levels of soluble tumor necrosis factor superfamily receptors in patients with hepatitis C virus infection and lymphoproliferative disorders. J Hepatol. 2001;34r(5):723‐729. 10.1016/s0168-8278(00)00063-5 11434619

[171] Wainstein MV , Mossmann M , Araujo GN , et al. Elevated serum interleukin‐6 is predictive of coronary artery disease in intermediate risk overweight patients referred for coronary angiography. Diabetol Metab Syndr. 2017;9:67. 28878828 10.1186/s13098-017-0266-5 PMC5585915

[172] Feinstein MJ , Nance RM , Drozd DR , et al. Assessing and refining myocardial infarction risk estimation among patients with human immunodeficiency virus: a study by the centers for aids research network of integrated clinical systems. JAMA Cardiol. 2017;2(2):155‐162. 28002550 10.1001/jamacardio.2016.4494 PMC5310962

[173] Maggi G , Bottelli R , Gola D , et al. Serum cholesterol and chronic hepatitis C. Ital J Gastroenterol. 1996;28(8):436‐440. 9032585

[174] Hansson GK , Hermansson A . The immune system in atherosclerosis. Nat Immunol. 2011;12(3):204‐212. 21321594 10.1038/ni.2001

[175] Chew KW , Bhattacharya D , McGinnis KA , et al. Short communication: coronary heart disease risk by Framingham risk score in hepatitis C and HIV/hepatitis C‐coinfected persons. AIDS Res Hum Retrovir. 2015;31(7):718‐722. 25858663 10.1089/aid.2014.0284 PMC4505770

[176] Ridker PM , Danielson E , Fonseca FA , et al. Rosuvastatin to prevent vascular events in men and women with elevated C‐reactive protein. N Engl J Med. 2008;359(21):2195‐2207. 18997196 10.1056/NEJMoa0807646

[177] Osibogun O , Ogunmoroti O , Michos ED , et al. A systematic review of the associations between HIV/HCV coinfection and biomarkers of cardiovascular disease. Rev Med Virol. 2018;28(1). 10.1002/rmv.1953 29135056

[178] Hou H , Wang C , Sun F , Zhao L , Dun A , Sun Z . Association of interleukin‐6 gene polymorphism with coronary artery disease: an updated systematic review and cumulative meta‐analysis. Inflamm Res. 2015;64(9):707‐720. 26174156 10.1007/s00011-015-0850-9

