# Supplementary material for: Inflammatory and cardiovascular diseases biomarkers in chronic hepatitis C virus infection: A review
Source: Clin Cardiol. 2019 Nov 30;43(3):222–34. doi: 10.1002/clc.23299 (PMC7068107; doi:10.1002/clc.23299)
Supplement: Supplementary file 1 — Appendix S1. Supporting Information. [file CLC-43-222-s001.docx]

**Supplementary Table 1: Biomarkers of Inflammation**

| Study (year) | Design | HCV study population: age in years; sex, %; race/ethnicity; country | Exclusion Criteria | Biomarkers evaluated | Sample Size, follow up time, calendar time | | Outcome | | | Ref. |
| --- | --- | --- | --- | --- | --- | --- | --- | --- | --- | --- |
| Larrea E *et al.* (1996) | Case-control | Age range: 16-75  Male: 67.4%  -  Spain | Non HCV chronic hepatitis | TNF-α | n=169, HCV: 129, Controls: 40  -  - | | - Mean serum TNF-α level significantly higher in untreated, NR and SVR HCV patients after 10-12 months IFN-α therapy vs. controls (untreated 166.28±33.21 pg/ml, NR 163.05±36.72 pg/ml and SVR 117.14±24.46 pg/ml vs. controls 19.28±5.20 pg/ml; *p* < 0.001)  -Serum TNF-α levels non significantly higher in NR vs. SVR (*p>*0.05) | | | 22 |
| Oliveira CP *et al.* (2013) | Case-control | Mean age: 52.9  Male: 54.8%  -  Brazil | Prior CVD, systemic HTN, CKD, cancer, alcohol abuse, pregnancy and chronic use of  lipid-lowering drugs immunosuppressants | TNF-α  IL-6  IL-10 | n=73, HCV: 62, Controls: 11  -  - | | -Serum TNF-α and IL-6 levels significantly higher in HCV vs. controls (TNF-α *p* = 0.002 and IL-6 *p* < 0.0001, respectively)  -No significant differences found in serum IL-10 levels in HCV vs. controls  -The ratios of serum TNF-α/IL-10 and IL-6/IL-10 (proinflammatory/anti-inflammatory) were significantly higher in HCV vs. controls (*p* < 0.01) | | | 31 |
| Falasca K *et al.* (2006) | Case-control | Mean age: 52.3  Male: 70%  -  Italy | HIV, other causes of liver dysfunction, chronic use of steatogenic or antiviral drugs, biochemical or clinical signs of advanced liver disease | IL-6  IL-10  TNF-α | n=60, HCV: 20, HBV: 20, Controls: 20  -  - | | -Mean serum IL-6 level significantly higher in HCV vs. controls (12.3 ± 19.1 pg/ml vs. 3.9 ± 11.5 pg/ml; *p* < 0.02)  -Mean serum TNF-α level significantly higher in HBV vs. HCV and controls (HBV 2.5 ± 3.0 pg/ml vs. HCV 0.4 ± 0.8 pg/ml and controls 0.5 ± 0.8 pg/ml; *p <* 0.005)  -Mean serum IL-10 level significantly higher in HCV vs controls (0.7 ± 1.5 pg/ml vs. 0.2 ± 0.2 pg/ml; *p <* 0.05) | | | 32 |
| Helaly GF *et al.* (2009) | Case-control | Age range: 35-60  Male: 72%  -  Egypt | GIB, malignancy  DM and alcohol intake | TNF-α  IL-6 | n=40, HCV: 25, Controls: 15  -  - | | -Mean serum TNF-α level significantly higher in HCV vs. controls (52.19±18.87 pg/ml vs. .> 2.3 pg/ml)  -Mean serum IL-6 level significantly higher in HCV vs. controls (8.25±4.99 pg/ml vs. 1.30±0.76 pg/ml; *p* = 0.001) | | | 33 |
| Khan S *et al.* (2011) | Case-control | -  -  -  India | Co-infection with hepatotropic viruses, alcohol and drug related hepatocellular injury | TNF-α  IL-6  IL-10  CRP | n=180, HCV: 60, HBV: 60 Controls: 60  -  - | | -Mean serum TNF-α level significantly higher in HCV vs. controls (24.88±1.55 pg/ml vs. 13.05±1.53 pg/ml; *p* < 0.05)  -Mean serum IL-6 level significantly higher in HCV vs. controls (51.68±2.36 pg/ml vs. 13.69±1.25 pg/ml; *p* < 0.05)  -Mean serum IL-10 levels significantly higher in HCV vs. controls (48.72±2.43 pg/ml vs. 12.88±1.60 pg/ml; *p* < 0.05)  -Mean serum CRP level significantly higher in HCV vs. controls (0.96±0.09 ng/ml vs. 0.32±0.04 ng/ml; *p* < 0.05)    -Positive linear correlation between HCV viral load and serum IL-6 and IL-10 levels observed (IL-6: *r* = 0.799, IL-10: *r* = 0.673; *p* <0.001)  -No correlation between HCV viral load and CRP levels noted | | | 34 |
| Tsui JI *et al.* (2009) | Case-control | Mean age: 59  Male: 80%  Non-white: 46%  United States of America | Absence of coronary heart disease | TNF-α  IL-6  CRP | n=981, HCV: 84, Controls: 897  Mean follow up: 4.1 years  2000-2002 | | -After adjustment mean serum CRP level significantly lower in HCV vs. controls (2.6 (95% CI 1.9, 3.5) mg/L vs. 4.4 (95% CI 4.1, 4.8) mg/L; *p* *<*0*.*01)  -After adjustment mean serum IL-6 level non-significantly higher in HCV vs. controls (3.6 (95% CI 3.0, 4.2) pg/ml vs. 3.2 (95% CI 3.0, 3.3) pg/ml; *p* = 0.13)  -After adjustment mean TNF-α level significantly higher in HCV vs. controls (7.1 (95% CI 5.8, 8.7) pg/ml vs. 4.8 (95% CI 4.6, 5.1) pg/ml; *p* < 0.01)  -HCV significantly associated with CHF hospitalizations vs. controls when adjusted for clinical factors and inflammatory markers (HR 2.05 (1.11–3.78); *p* = 0.02) | | | 35 |
| Migita K *et al.* (2006) | Case-control | Mean age: 63.4  Male: 45.5%  -  Japan | Fluctuations in  LFTs, auto-immune or alcoholic liver disease, metabolic disease, other co-infections, CKD | IL-6  sIL-6R  sgp130 | n=100, HCV: 77, Controls: 23  -  - | | - Serum IL-6 levels significantly higher in CHC vs. controls (*p* < 0.04) and serum IL-6 significantly higher in cirrhotics vs. CHC (*p* < 0.01)  -Serum IL-6R level significantly higher in CHC vs. controls (*p* < 0.01)  -No difference found in sgp130 levels between HCV and controls | | | 36 |
| Zekri AR *et al.* (2005) | Case-control | -  Male: 75.3%  -  Egypt | - | IL-6  IL-6R  IL-10  TNF-α  TNF-RI  TNF-RII | n= 93, HCC/HCV+: 33, Chronic liver diseases (CLD) HCV+: 20, Asymptomatic HCV (ASC) : 20, Controls: 20  -  2000-2001 | | -Mean serum IL-6 level significantly higher in ASC vs. CLD vs. HCC vs. controls (ASC 6.4±1.9 ng/ml vs. CLD 5.2±1.68 ng/ml vs. HCC 4.71±2.49 ng/ml vs. controls 4.82±2.62 respectively; *p* = 0.032  -Mean serum IL-6R level significantly higher in HCC, CLD and ASC vs. controls (HCC 56.48±17.97 ng/ml, CLD 54.47±16.72 ng/ml and ASC 54.13±16.35 ng/ml vs. controls 19.62±5.52 ng/ml respectively; *p* <0.001)  -Mean serum IL-10 level non-significantly higher in HCC and CLD vs. ASC and controls (HCC 9±26.07 pg/ml and CLD 6.59±7.57 pg/ml vs. ASC 3.96±2.24 pg/ml and controls 3.13±0.79; *p* >0.05)  -Mean serum TNF-α level significantly higher in ASC vs. HCC, CLD and controls (ASC 811±5.8 ng/ml vs. HCC 4.77±3.2 ng/ml, CLD 5.53±1.9 ng/ml and controls 58±196; *p* <0.001)  -Mean serum TNF-α RI level significantly higher in HCC and CLD vs. ASC and controls (HCC 1.27±0.98 ng/ml and CLD 1.87±1.50 ng/ml vs. ASC 0.56±0.23 ng/ml and controls 0.29±0.16 ng/ml; *p* < 0.001)  -Mean serum TNF-α RII level significantly higher in HCC and CLD vs. ASC and controls (HCC 7.89±3.57 ng/ml and CLD 9.16±4.26 ng/ml vs. ASC 3.42±1.72 ng/ml and controls 2.17±1.29 ng/ml; *p* < 0.001) | | | 37 |
| Malaguarnera M *et al.* (1997) | Case-control | Mean age: 56.8±9.6  Male: 41.7%  -  Italy | - | IL-6 | n= 63, HCV: 36, Controls: 27  -  - | | -Mean serum IL-6 level significantly higher in HCV vs. controls (21.6±2.8 pg/ml vs. 9.7±5.9 pg/ml; *p* < 0.001)  -Positive linear correlation observed between IL-6 levels and HAI score in HCV (*r* =0.735; *p* < 0.001) | | | 38 |
| Costantini S *et al.* (2010) | Case-control | Mean age: 65.9  Male: 48.3%  -  Italy | HCC | IL-6 | n= 80, CHC: 30, HCV-related cirrhosis: 30, Controls: 20  -  - | | -Mean serum IL-6 level significantly higher in CHC vs. controls (*p* = 0.0032)  -Mean serum IL-6 level significantly higher in HCV-related cirrhosis patients vs. controls (*p* = 0.0024) | | | 39 |
| Capone F *et al.* (2010) | Case-control | Mean age: 70  Male: 69.2%  -  Italy | - | IL-10  IL-6 | n= 46, HCV-related HCC: 26, Controls: 20  -  - | | -Mean serum IL-10 and IL-6 levels significantly higher in HCV-related HCC patients vs. controls; IL-10 *p* = 0.0214, IL-6 *p* = 0.0183)  -Mean serum IL-6 level correlated significantly with large tumor size (r> 0.83; *p* < 0.05) | | | 40 |
| Oyanagi Y *et al.* (1999) | Case-control | -  -  -  Japan | Administration of therapeutic agents which would influence serum IL-6 levels,  interferon therapy. | IL-6 | n= 89, HCV: 63, Controls: 26  -  - | | -Mean serum IL-6 level significantly higher in HCV vs. controls (11.5±14.6 pg/ml vs. 6.5±8.9 pg/ml; *p* < 0.01) | | | 41 |
| Lapinski TW *et al.* (2001) | Case-control | Age range: 18-53  Male: 67.6%  -  Poland | - | IL-6 | n= 55, HCV: 34, Liver cirrhosis (HBV-, HCV-): 9, Controls: 12  -  - | | -Mean serum IL-6 level significantly higher in HCV vs. controls (1.393±1.247 pg/ml vs. 0.600±0.760 pg/ml; *p* <0.001) | | | 42 |
| Lecube A *et al.* (2006) | Case-control | Mean age: 48.5  Male: 35.7%  -  Spain | DM, alcohol consumption, corticosteroid, previous treatment with interferon, HIV, hemochromatosis, chronic pancreatitis, neoplasia, CKD | IL-6  TNF-α  sTNFR-I  sTNFR-II | | n= 42, HCV: 28, Controls: 14  -  - | | - Mean serum IL-6 level significantly higher in HCV vs. controls (3.78±1.45 pg/ml vs. 2.07±1.02 pg/ml; *p* = 0.002)  - Mean serum TNF-α level significantly higher in HCV vs. controls (6.47±3.44 pg/ml vs. 2.71±3.06 pg/ml; *p* = 0.007)  - Mean sTNFR-I level significantly higher in HCV vs. controls (1,739.58±347.35 pg/ml vs. 1,494.16±270.63 pg/ml; *p* = 0.040)  - Mean serum sTNFR-II level significantly higher in HCV vs. controls (2,999.16±785.55 pg/ml vs. 2,417.50±815.59 pg/ml; *p* = 0.046) | | 43 |
| Sandler NG *et al.* (2011) | Retrospective observational cohort | Mean age: 49.9  Male: 35.7%  -  United States of America | Underlying intestinal disease, | IL-6  sCD14 | | n= 151, HCV: 63, SVR: 27, HBV: 21, Controls: 67  -  - | | - Serum IL-6 and sCD14 levels were significantly higher in HCV vs. controls  -Serum CD14 levels was significantly higher in progressors than non-progressors (2.14 × 10^6^ vs 1.81 × 10^6^ pg/ml; *p*=0.003)  -Levels of sCD14 did not change appreciably with treatment | 44 | |
| Grungreiff K *et al.* (1999) | Case-control | -  -  -  Germany | HIV, autoantibodies, active infection  . | IL-6 | | n= 28, HCV: 16, Complete responders: 4, Partial responders: 6, NR: 6 Controls: 12  -  - | | -Mean serum IL-6 level higher in HCV vs controls (7.10 ± 2.20 vs 0.65 ± 0.13)  - Serum IL-6 levels in complete and partial responders  showed a steady decline with therapy | | 46 |
|  |  |  |  |  | |  | |  | |  |
| Cotler SJ *et al.* (2001) | Case-control | - Mean age: 40.7  -  -  United States of America | - | IL-6  TNF-α | | n= 95, HCV: 45,  Previous NR: 18  Treatment naïve: 27  Controls: 50 | | - Mean serum TNF-α levels not significantly different in HCV vs. controls (2.37±0.7 pg/ml vs. 2.26±0.3 pg.ml)  -Mean serum IL-6 level higher in NR and treatment naïve HCV vs controls (NR 4.88 ± 2.6, treatment I 6.25±1.6 vs controls undetectable)  -Baseline IL-6 levels increased rapidly after a single  IFN treatment, change in HCV titers was not significantly associated with pretreatment IL-6 level or with the change in IL-6 levels | | 48 |
| Hung C-H *et al.* 2009 | Case-control | Mean age: 51.9±12  Male: 46%  -  Taiwan | HBV, HIV,  autoimmune hepatitis, primary biliary cirrhosis, sclerosing cholangitis, Wilson’s disease, a1-antitrypsin deficiency,  decompensated LC, alcohol abuse, psychiatric condition, HCC | IL-6  TNF-α  IL-10 | | n= 121, HCV:81, Controls: 40  -  2006-2007 | | -Mean serum IL-6 level significantly higher in HCV vs. controls (5.5 ±3.5 vs. 3.1 ±2.3; *p=*0.001)  -Mean serum TNF-α level significantly higher in HCV vs. controls (2.1±0.8 vs. 1.1±0.4; *p*= 0.004)  -Mean serum IL-10 level significantly higher in HCV vs. controls. (3.0±1.8 vs. 1.1±1.8; *p*= 0.048) | | 49 |
| Shive CL *et al.* (2018) | Clinical trial | Mean age: 53  Male: 27.8%  Caucasian: 67%, African American: 46.2%  Italy | HBV, history of HAV, HAV or HBV vaccine, systemic antineoplastic treatment, recent therapeutic radiation | IL-6  sCD14  sCD163 | | n= 49, HCV: 15, HIV: 24, Controls: 10  -  2001-2006 | | - Median serum IL-6, sCD14, and sCD163 levels significantly higher in HCV vs. controls (IL-6, *p* <0.001; sCD14, *p* = 0.03; sCD163, *p* = 0.0002) | | 52 |
| Cua IH *et al.*  (2007) | Case-control | Mean Age: 40.6 ±8.6  Male: 100%  -  Australia | HCV genotype 2, 4, 5 and 6, females, decompensated LC, HVB, HIV, autoimmune hepatitis, drug-induced steatohepatitis, primary biliary cirrhosis, primary sclerosing cholangitis, haemochromatosis, Wilson’s disease and 1-antitrypsin deficiency | IL-6  TNF-α | | n=229 HCV: 154, Controls:75  -  - | | -Median serum IL-6 level significantly higher in HCV vs. controls 2.42 [IQR 0.91- 6.30] pg/ml vs. 1.15 [IQR 0.45- 2.82] pg/ml; *p=* 0.001  - Median serum TNF-α level significantly higher in HCV vs. controls 1.28 [IQR 0.43- 1.90] pg/ml vs. 0.60 [IQR 0.21- 1.19] pg/ml; *p<*0.001) | | 53 |
| Grungreiff K *et al.* (2009) | Case-control | Mean age: 53.8  Male: 36%  -  Germany | HIV, autoantibodies, active infection | IL-6 | n=80, HCV: 50, Controls: 30  -  - | | -Mean serum IL-6 significantly higher in female and male HCV vs. female and male controls (female HCV 5.5±1.76 pg/ml and male HCV 6.4±3.22 pg/ml vs female controls1 0.4±0.29 pg/ml and male controls 2.5±1.51 pg/ml; *p* < 0.05) | | | 54 |
| Zuwała-Jagiełło J *et al.* (2011) | Case-control | Mean age: 58  Male: 63.4%  -  Poland | Antioxidant  Drug therapy, DM, CKD,  cardiovascular disease, HCC, recent alcohol use GIB or blood transfusion. | CRP  TNF-α  IL-6 | | n=114, HCV cirrhosis: 41, Alcoholic cirrhosis: 43, Controls: 30  -  1998-2003 | | -Mean serum TNF-α level significantly higher in decompensated HCV cirrhosis and compensated HCV cirrhosis vs. controls (decompensated HCV cirrhosis 42.4 ± 4.80 pg/ml and compensated HCV cirrhosis 40.0 ± 2.3 pg/ml vs. controls 33.11 ± 2.06 pg/ml; *p* < 0.05)  -No difference in mean serum IL-6 levels in HCV cirrhosis vs. controls (9.01 ± 1.18 pg/mL vs. 6.99 ± 0.98 pg/mL; *p* > 0.05)  -Mean serum CRP level significantly higher in decompensated HCV cirrhosis and compensated HCV cirrhosis vs. controls (decompensated HCV cirrhosis 6.80 ± 1.20 mg/L and compensated HCV cirrhosis 5.96 ± 1.05 mg/L vs. controls 1.27 ± 0.64 mg/L; *p* < 0.05) | | 57 |
| Mourtzikou A *et al.* (2014) | Case-control | -  Male: 80%  -  Greece | HIV, other causes of chronic liver disease, alcohol consumption, immunosuppressive or antiviral therapy. | IL-6  TNF-α  IL-10 | | n= 120, HBV: 50, HCV: 40, Controls: 30  -  - | | -No significant difference in mean serum IL-6 level in HCV vs. controls (2.501 pg/ml vs. 0.744 pg/ml; *p* > 0.05)  -Mean serum TNF-α level significantly higher in HCV vs. controls (7.519 pg/ml vs. 1.323 pg/ml; *p* < 0.05)  -Mean serum IL-10 level significantly higher in HCV vs controls  (12.431 pg/ml vs 2.130; *p* < 0.05) | | 58 |
| Akcam FZ *et al.* (2012) | Case-control | Mean age:48.6  Male: 52%  -  Turkey | Previous antiviral therapy | TNF-α  IL-10 | | n=80, HCV: 25, HBV: 25, Controls: 30  -  2007-2009 | | -Mean serum TNF-α level significantly higher in HBV and HCV vs. control (HBV 6.58±7.26 pg/ml, HCV 5.05±3.42 pg/ml vs. controls 3.04±2.09 pg/ml; *p* = 0.035)  -Mean serum IL-10 level significantly higher in HBV and HCV vs. controls (HBV 8.77±5.37 pg/ml, HCV 12.98±21.10 pg/ml respectively vs. controls 0.05±0.02 pg/ml; *p* = 0.002) | | 59 |
| Talaat RM *et al.* (2010) | Case-control | -  Male: 81.7%  -  Egypt | HBV | TNF-α | | n= 102, HCV: 82, Controls: 20  -  - | | -Serum TNF-α levels significantly higher in HCV vs. controls (*p* < 0.001)  - Gradual elevations in TNF- α levels in HCV+ with increasing severity of cirrhosis (p<0.001) | | 60 |
| Abdel-Latif MS *et al.* (2015) | Case-control | -  -  -  Egypt | DM, HBV, Schistosomiasis, HCC | TNF-α | | n= 40, HCV: 25, Controls: 15  -  - | | -Mean serum TNF-α level significantly higher in HCV vs. controls (0.36±0.03 pg/ml vs. 0.15±0.01 pg/ml; *p* <0.001) | | 61 |
| Kallinowski B *et al.* (1998) | Case-control | Age range: 24-66  Male: 64.8%  -  Germany | HBV, HIV, autoimmune hepatitis | TNF-α  TNFR-p55  TNFR-p75 | | n=150, HCV: 105, Controls: 45  -  - | | -Mean serum TNF-α level significantly higher in HCV vs. controls (83.86±91.7 pg/ml vs. 18.86± 8.4 pg/ml; *p* < 0·001)  -Mean serum TNFR-p55 and TNFR-p75 levels significantly higher in HCV vs. controls (HCV: TNFR-p55 1.46±0.40 ng/ml and TNFR-p75 6.46±2.4 ng/ml vs. controls: TNFR-p55 0.96±0.20 ng/ml and TNFR-p75 2.96 ± 0.60 ng/ml; *p* < 0·0001)  -Mean serum TNFR-p55 and TNFR-p75 levels significantly lower in patients with HCV and minimal to moderate necroinflammatory activity vs. HCV patients with moderate to severe necroinflammatory activity (mild/moderate TNFR-p55 1.36±0.3 ng/ml and TNFR-p75 5.66±1.8 ng/ml vs. moderate/severe TNFR-p55 1.66±0.5 ng/ml and TNFR p-75 8.16±2.8 ng/ml; *p* < 0·01)  -Linear correlation observed between serum TNFR-p55 and TNFR-p75 levels with HAI (*r* = 0.28 and *r* = 0.50 respectively; *p* < 0·01 for both)  -No significant differences observed in pre-treatment serum levels of TNF-α, TNFR-p55 and TNFR-p75 among responders and non-responders to IFN-α therapy | | 62 |
| Jia HY *et al.* (2002) | Case-control | Age range: 18-58  Male: 78.4%  -  China | Neurological or cardiovascular disease, CKD, co-infection with hepatotropic viruses, interferon or immunosuppressive therapy | TNF-α | | n= 61, CHC: 27, ASC HCV: 24, Controls: 10  -  1999-2000 | | -No difference in mean serum levels of TNF-α and IL-10 between ASC HCV and controls (*p*<0.05)  -Mean serum levels of TNF-α and IL-10 significantly higher in CHC vs. controls (TNF-α 35.09±3.21 pg/ml vs. 22.39±1.98 pg/ml; *p* <0.05 and IL-10 48.87± 7.50 pg/ml vs 17.70 ±1.32; *p* < 0.05)  - Mean serum levels of Il-10 and TNF-α significantly higher in NR and partial responders vs SVR (*p*<0.005)  -Mean serum levels of IL-10 and TNF-α levels decreased significantly with treatment (*p*<0.005) in all groups | | 65 |
| Sayed-Ahmed L *et al.* (2010) | Case-control | Mean age: 44.2  Male: 75%  -  Egypt | HBV, HIV, DM, family history of DM, antiviral or corticosteroid therapy,  malignancies, liver fibrosis, LC, active infections, CKD, respiratory or cardiac dysfunction, obesity | TNF-α | | n= 64, HCV: 44, Controls: 20  -  - | | -Mean serum TNF-α level significantly higher in HCV vs. controls (87.6±31.2 pg/ml vs. 22.7±12.1 pg/ml; *p* < 0.001)  -Serum TNF levels correlated with insulin resistance (*r*=0.890; *p*=0.001) | | 69 |
| Toyoda M *et al.* (2000) | Case-control | Mean age: 48.7  Male: 68.3%  -  Japan | HBV, RA, inflammatory bowel syndrome, myelogenous leukemia, SLE | TNF-α | | n= 71, HCV: 41, Controls: 30  -  - | | -Mean serum TNF-α level significantly higher in HCV vs. controls (70.8±82.2 pg/ml vs. 28.2±24.6 pg/ml; *p* < 0.01)  - Serum TNF-α levels showed no correlation with histological activity or serum HCV RNA levels  -Serum TNF-a levels increased in 7 patients (54%) among CR, and in 10 patients (63%) among NR | | 70 |
| Kishihara Y *et al.* (1996) | Case-control | Mean age: 50  Male: 60.9%  -  Japan | HBV, HIV, autoantibodies, active infection, RA, inflammatory bowel syndrome, myelogenous leukemia, DM, SLE | TNF-α | | n= 79, HCV: 69, Controls: 10  -  - | | -Mean serum TNF-α significantly higher in HCV vs. controls (272.7±331.3 pg/ml vs. 48.8±15.4 pg/ml; *p* < 0.05) | | 71 |
| Raghuraman S *et al.* (2005) | Case-control | Mean age: 47.5  Male: 58.8%  -  India | Antiviral or immunosuppressive therapy, immunological disorder, | TNF-α | | n=46, HCV: 17, HIV: 19, Controls: 10  -  - | | -Mean serum TNF-α level significantly higher in HCV vs. HIV and controls (HCV 73.11±63.41 pg/ml vs. HIV 62.76±27.47 pg/ml and controls 21.97±4.86 pg/ml; *p* = 0.01) | | 72 |
| Valenti L *et al.* (2005) | Case-control | Mean age: 50.5  Male: 59%  -  Italy | HBV, HIV, HCC, alcohol abuse, previous interferon therapy | TNF-α  TNFR-p75 | | n=275, HCV: 186, Controls: 89  -  1998-2003 | | -Prevalence of detectable serum TNF-α levels (>0.5 pg/ml) significantly higher in HCV vs. controls (46% (87/186) vs 10% (9/89); *p* < 0.0001)  -Mean serum TNFR-p75 level significantly higher in HCV vs. controls (3890±1168 pg/ml vs. 2288±564 pg/ml; *p* < 0·0001) | | 73 |
| Nelson DR *et al.* (1997) | Case-control | Age range: 18-76  Male: 43.4%  -  United States of America | Other causes of chronic liver diseases | TNF-α  sTNFR-I  sTNFR-II | | n= 86, HCV: 53, Controls: 33  -  - | | -Mean serum TNF-α level significantly higher in HCV vs. controls (9.62±6.01 pg/ml vs. 3.66±1.23 pg/ml; *p* < 0.001)  - Mean sTNFR-I level significantly higher in HCV vs. controls (3,325±1,267 pg/ml vs. 1,856±423 pg/ml; *p* < 0.001)  - Mean serum sTNFR-II level significantly higher in HCV vs. controls (1,291±651 pg/ml vs. 864±207 pg/ml; *p* < 0.001)  -Linear correlation observed between TNF-α levels and HAI (*r* = 0.39; *p* = 0·015) | | 75 |
| Glowacki MK *et al.* (2014) | Case-control | Mean age: 39.3  -  -  Poland | Previous antiviral therapy, pregnancy, obesity, alcohol  abuse, autoimmune diseases, NAFLD,  inflammatory  liver diseases, inflammatory con-  ditions, chronic severe diseases, malignancy, DM | TNF-α | | n= 60, HCV: 30, Controls: 30  -  - | | -Mean serum TNF-α level significantly higher in HCV vs. controls (11.0±19.3 pg/ml vs. 3.3±2.8 pg/ml; *p* = 0.041) | | 76 |
| Riordan SM *et al.* (2006) | Case-control | Mean age: 39  Male: 77.8%  -  Australia | HIV, HBV, alcoholic liver diseases, NAFLD, alcohol use, infection, treatment with antibiotics, probiotics, lactulose, immunomodulatory drugs or GIB, CKD | TNF-α | | n= 50, HCV: 18, Controls: 32  -  - | | -Mean serum TNF-α level significantly higher in HCV vs. controls (*p* <0.0005) | | 77 |
| Zylberberg H *et al.* (1999) | Case-control | Mean age: 48  Male: 68.3%  -  France | HIV, organ transplantation, hemodialysis, hemopathy or drug-related immunosuppression, previous antiviral therapy | TNF-α  sTNFR-p55  sTNFR-p75 | | n= 154, HCV: 60, HBV: 34, Controls: 60  -  - | | -Mean TNF-α level higher in HCV vs controls (50.4±4.5 pg/ml vs undetectable)  -Mean serum levels of TNFR-p55 and TNFR-p75 significantly higher in HCV vs. controls (HCV: TNFR-p55 2.88±0.14 ng/ml and TNFR-p75 9.54±0.58 ng/ml vs. controls: TNFR-p55 1.30±0.05 ng/ml and TNFR-p75 4.19 ± 0.16 ng/ml; *p* = 0·0001)  -TNF-α levels increased with Metavir activity score (p= 0.03)  -No correlation between serum HCV RNA levels and levels of sTNF-R55 (*p*=0.92, sTNF-R75 (*p*=0.92) and TNF- α (*p*=0.81)  - No significant variation in sTNF-R levels during therapy  - No significant differences in levels of sTNF-R55, sTNF-R75, TNF-a in NR vs SVR groups | | 78 |
| Itoh Y *et al.* (1999) | Case-control | -  -  -  Japan | Heavy alcohol consumption, obesity, coinfection of other hepatotropic viruses. | sTNFR-p55  sTNFR-p75 | | n= 104, HCV: 84 (ASC:35, SVR: 24, NR:25), Controls: 20  -  1992-1995 | | -Median sTNFR-p55 level significantly higher in ASC HCV vs. controls (1.06 (range 0.6-1.46) ng/ml vs. 0.84 (range 0.56-1.36) ng/ml; *p* =0.0048)  -Median sTNFR-p55 level significantly higher in CHC vs. ASC HCV (1.28 (range 0.86-1.94) ng/ml vs. 1.06 (range 0.6-1.46) ng/ml; *p* = 0.002)  -Median sTNFR-p75 level significantly higher in ASC HCV vs. controls (2.55 (range 1.64-5.62) ng/ml vs. 1.51 (range 0.56-3.16) ng/ml; *p* =0.0031)  -Median sTNFR-p75 level significantly higher in CHC vs. ASC HCV (3.70 (range 0.44-6.72) ng/ml vs. 2.55 (range 1.64-5.62) ng/ml); *p* = 0.0067)  -Significant correlation found between levels of sTNFR p55 and sTNFR p75 and total HAI score (r = 0.561; *p<*0.0001, *r*=0.598; *p<* 0.0001)  -Levels of sTNFR-p75 significantly decreased 12  months after IFN therapy in SVR group (*p* <0.0002) but not in NR group (*p* = 0.8710) | | 79 |
| Kakumu S *et al.* (1997) | Case-control | Mean age: 57  Male: 70.4%  -  Japan | Co-infection with other hepatotropic viruses. | IL-10  sTNFR-p55  sTNFR-p75 | | n= 82, HCV: 71(ASC:10, CHC: 28, LC:22, HCC: 11), Controls: 11  -  - | | -Mean serum IL-10 level significantly higher in all HCV groups vs. controls (*p* < 0.05)  -Mean sTNFR-p55 significantly higher in HCC and LC vs. controls (HCC 1,741±460 pg/ml and LC 2,071±580 pg/ml vs. controls 1,1170±291 pg/ml; *p* < 0.05)  -Mean sTNFR-p75 significantly higher in LC and HCC vs. CHC, ASC and controls (LC 5.4±1.5 ng/ml and HCC 6.0±3.0 ng/ml vs. CHC 3.0±1.0 ng/ml, ASC 4.1±1.3 ng/ml and controls 2.0±0.4 ng/ml; *p* < 0.05)  - No significant correlation with serum cytokines and HCV RNA  -Treatment decreased serum levels of IL-10 but not levels sTNFR-p75 and sTNFR-p77 | | 80 |
| Verma V *et al.* (2008) | Case-control | -  Male: 71.1%  -  India | HBV, HIV | IL-10  sTNFR-p75 | | n= 110, HCV: 90 (HCC: 8, LC: 20, CHC: 62), Controls: 20  -  2004-2006 | | -Mean serum IL-10 level significantly higher in HCC, LC and CHC vs. controls (HCC 127.33±106.61 pg/ml, LC 73.73±39.30 and CHC 84.51±54.26 pg/ml vs. controls 21.59±8.67 pg/ml; *p* < 0.001)  -Mean sTNFR-p75 significantly higher in HCC, LC and CHC vs. controls (HCC 80.50±8.33 ng/ml, LC 60.35±27.61 ng/ml and CHC 18.48±16.68 ng/ml vs. controls 4.33±0.79 ng/ml; *p* <0.001) | | 81 |
| Farag NH *et al.* (2011) | Case-control | Mean age: 37  -  -  Egypt | LC, HCC, hepatic encephalopathy, interferon therapy, drug or alcohol use, severe or untreated psychiatric disorder, DM, HIV, HBV, parasitic infections | sTNFR-p75  sCD14 | | n= 25, HCV: 11, Controls: 14  -  - | | -Mean serum sTNFR-p75 level significantly higher in HCV vs. controls (10.2±0.83 ng/ml vs. 6.8±0.57 ng/ml; *p* = 0.002)  -No difference in mean serum sCD14 level of HCV vs. controls (69.5±5.2 ng/ml vs. 67.6±13.3 ng/ml; *p* > 0.05) | | 82 |
| Markowtiz M *et al.* (2016) | Cross-sectional | Mean Age: 42.1  Male: 76.6%  Caucasian: 65.0%  USA | HIV, Coagulopathy | sCD14 | | N=192, HCV: 144 (Active IVDU ([Group 1]:48, Ceased IVDU 1-2 months prior [Group 2]: 48, Ceased IVDU] 3-4 months prior [Group 3: 48), Controls: 48  -  - | | - Mean serum sCD14 level significantly higher in HCV viremic individuals vs. controls (Group 1:1601 ± 296 ng/mL, Group 2 1796 ± 276 ng/mL and Group 3: 1730 ± 243 ng/mL vs. Controls: 1388 ± 206; *p*<0.05) | | 83 |
| Lee SH *et al.* (2010) | Case-control | Mean age: 53.4  Male: 50%  -  Korea | LC, HCC | IL-6 | | n= 99, CHC: 28, ALD: 22, NAFLD: 24, Controls: 25  -  2006-2007 | | -Serum IL-6 levels significantly higher in CHC vs. controls (358.60±242.11 pg/ml vs. 1.25±0.68 pg/ml; *p* < 0.05) | | 84 |
| Han ZQ *et al.* (2015) | Case-control | Mean age: 36  Male: 69.4%  -  China | HIV, HBV, recent antiviral or immunomodulatory therapy | IL-6  IL-10 | | n= 93, CHC: 72, Controls: 21  -  2012-2014 | | -No difference in mean serum IL-6 levels between HCV and controls  -Serum IL-10 levels significantly higher in HCV vs. controls (16.19±5.89 pg/mL vs. 32.21±24.85 pg/mL; *p* = 0.0035)  - Serum IL-10 levels higher in HCV genotype 1b vs genotype 2a (37.16 ± 27.42 pg/mL vs 21.67 ± 13.49 pg/mL; *p* = 0.0079) | | 85 |
| Priimägi L *et al.* (2005) | Case-control | -  -  -  Estonia | - | IL-10 | | n= 82, HBV: 15, HCV: 21, HBV+HCV: 26, Controls: 20  -  2000-2003 | | -Mean serum IL-10 level significantly higher in HCV vs. controls (32.0±9.9 pg/mL vs. 10.8±7.8 pg/mL; *p* < 0.05)  -No correlation between cytokine levels and HCV RNA levels found | | 86 |
| Mishra PK *et al.* (2010) | Case-control | -  -  -  India | Co-infection with hepatotropic viruses  hepato-cellular injury due to alcohol and drug intake | IL-6  IL-10  TNF- α | | n= 180, HBV: 60, HCV: 60, Controls: 60  -  - | | -Serum IL-6, IL-10 and TNF- α levels significantly elevated in HCV patients vs. controls (*p* < 0.05)  -HCV viral loads correlated with IL-6 (*r* = 0.799; *p*< 0.001) and IL-10 levels (r = 0.673; *p*< 0.001) | | 87 |
| Fan XG *et al.* (1998) | Case-control | Mean age: 32 (20-47)  Male: 63.6%  -  China | Co-infection with hepatotropic,  hepatocellular  injury, due to alcohol, drugs and autoimmune disease, recent immunoregulatory therapy | IL-10 | | n= 39, HCV: 18, HBV: 10, Controls: 11  -  - | | -IL-10 levels significantly increased in HCV vs controls (50.30± 19.59 vs 17.87±9.49 pg/ml; *p*<0.001) | | 88 |
| Marín-Serrano E *et al.* (2006) | Case-control | Mean age: 38 (35-41)  Male:74%  -  Spain | Active alcohol or drug dependence, HIV, HBV, infectious, autoimmune, tumoural, biliary, or vascular‐associated liver disease | IL-10 | | n=42, HCV:27, Controls: 15  -  - | | -Baseline mean serum IL-10 level was significantly higher in HCV vs controls (*p*<0.01)  -Baseline mean serum IL-10 level was significantly higher in NR vs SVR (12 (0-35) pg/mL vs 21 (0-62) pg/mL; *p*<0.001) | | 89 |
| Sousa GM *et al.* (2012) | Case-control | Mean age: 47.8  Male: 46.7%  -  Brazil | HIV, HBV, HTLV-I/II and the presence of bacterial or parasitic diseases | IL-6 | | n= 88, CHC: 60, Controls: 28  -  - | | -No difference in median serum IL-6 levels observed between HCV and controls (3.0 [IQR 2.2-4.4] pg/ml vs. 3.0[IQR 2.8-4.8] pg/ml; *p* > 0.05)  -No difference noted in serum IL-6 levels between CHC with mild necroinflammatory activity (A0/A1) and controls (*p* > 0.05)  -Serum IL-6 levels significantly higher in CHC with moderate liver necroinflammatory activity (A2) vs. CHC with mild necroinflammatory activity (A0/A1) and controls (*p* < 0.05) | | 91 |
| Müller C *et al.* (1992) | Case-control | Mean age: 54±11  Male: 53.9%  -  Austria | HCC, upper GIB, infection, fever, malnutrition, CKD, | IL-6 | | n= 100, CHC: 13, HBV: 14, Alcoholic cirrhosis: 22, Primary biliary cirrhosis: 6, Controls: 45  -  - | | -No significant difference in spontaneous or LPS induced mean serum IL-6 levels in HCV vs controls (spontaneous 150 ± 32 U/ml vs. LPS-induced 89 ±22, 860 ± 92 vs controls 672 ± 151) | | 92 |
| Bruno C *et al.* (2011) | Case-control | Mean ag: 56±8  Male: 55.1%  -  Italy | HCC, Autoimmune diseases, alcohol abuse, and drug-induced liver injury, evidence of chronic or acute infective processes | IL-10 | | n=69, HCV: 49, Controls: 20 | | -No significant difference in mean serum IL-10 level in HCV vs controls (16.1 ± 2.1 pg/mL vs 15.4 ±1.9 pg/ml; *p*=0.172)  -Mean serum IL-10 levels significantly higher in patients with higher HAI score (9-18) vs lower HAI score (1-8) (17.2 ±2.4 pg/ml vs 15.4 ±0.9 vs pg/ml; *p*<.001) | | 93 |
| Zografos TA *et al.* (2008) | Case-control | Mean age: 38.8±14.3  Male: 67.5%  -  Greece | - | TNF-α | | n= 185, CHC: 83, CHB: 59, Controls: 43  -  - | | -Mean serum TNF-α level significantly higher in CHB vs. controls (26.8 ± 83.7 pg/ml vs. 1.14±2.5 pg/ml; *p* = 0.02)  -Mean serum TNF-α levels did not differ at the end of treatment, or after 6 months of follow-up vs. baseline levels | | 96 |
| Moura TCF *et al.* (2019) | Cross-sectional | -  -  -  Brazil | Coinfection with HDV,  HIV-1, antiviral  therapy | CRP | | n= 485, HBV: 74, HCV: 111, Controls: 300  -  2013-2016 | | -Serum CRP concentration significantly higher in HBV vs. HCV (*p* = 0.021)  -Serum CRP concentration significantly lower in HCV vs. controls (*p* = 0.001) | | 97 |
| Ufearo H *et al.* (2010) | Cross-sectional | Median age: 48  Male: 71%  African American: 100%  - | Non-African American, acute illness | CRP | | n= 134, HCV+: 98, HCV-: 36  -  - | | -Mean serum CRP level significantly higher in HCV- vs. HCV+ (2.4 (95% CI 1.9, 3.2) mg/l vs 1.3 (95% CI 0.9, 2.1) mg/l; *p=*0.026) | | 98 |
| Adinolfi LE *et al.* (2013) | Case-control | Median age (range): 54 (22–70)  Male: 51  –  Italy | Acute myocardial infarction, severe cardiac valvular disease, CHF, , left ventricular akinesis or aneurysm, inflammatory valvulitis, nonischemic cardiomyopathies | CRP | | n = 803, HCV: 326, Controls: 477  –  2005–2011 | | -Median serum CRP level significantly higher in HCV-+ vs. controls (1.5 ± 1.5 mg/dl vs. 0.72 ± 0.58 mg/dl; *p*= 0.001)  - Viral load independently associated with carotid atherosclerosis (*p* < 0.0001) | | 100 |
| Yilmaz S *et al.* (2007) | Cross-sectional | Mean age: 40.1  Male: 80.0%  -  Turkey | Antiviral or immunomodulatory therapy, excessive alcohol consumption, CKD | hsCRP | | n=98, HCV:42, HCV LC: 28, Controls:28  -  - | | -Mean hsCRP level significantly higher in HCV+ vs. controls (HCV: 0.35 ± 0.72 mg ⁄ dl and HCV LC: 3.62 ± 4.36 mg ⁄ dl vs. Controls: 0.11± 0.13 mg ⁄ dl; *p* < 0.002) | | 101 |
| Alyan O *et al*. (2008) | Case-control | Mean age: 61.2  Male: 76.3  -  Turkey | HBV, LC, acute or chronic inflammatory disease, immunological disease, and a history or the presence of neoplastic disease | CRP | | n = 364, HCV: 139, Controls: 225  -  - | | - Mean serum CRP levels significantly higher in HCV+ vs controls (2.073±1.358 mg/l vs 1.190±1.005 mg/l; p<0.001)  -CRP level found to be independent predictor of severity of CAD: OR 0.277 (95% CI 0.096, 0.459); *p=*0.003 | | 102 |
| Zuwala-Jagiello J *et al.* (2015) | Case-control | Median Age: 56 (21-74)  Male: 44.2%  -  Poland | Antioxidant drugs, CKD, DM, cardiovascular disease, CHF, HCC, GIB, bacterial infection, recent blood transfusion | hsCRP  TNF- α  hs-TnT | | n=160, HCV: 120, HCV LC: 88, Controls: 40  -  - | | -Median serum hsCRP level significantly higher in HCV patients child Pugh class A, B and C vs controls (class A 3.8 [IQR 3.1–7.0] mg/L;  *p <*0.05, class B 5.2 [IQR 4.9–7.7];  *p*<0.01, class C 6.3 [IQR 5.8–11.0];  *p*<0.01 vs controls 1.05 [IQR 0.58–2.5] mg/L)  -Median serum TNF-𝛼 levels were higher in child Pugh class B and C patients vs controls: (class B 42.0 [IQR 37.6–47.2] pg/ml; *p <*0.05, class C 58.7 [IQR 48.7–64.0] vs 25.0 [IQR 20.5–30.2] pg/ml; *p*<0.01)  - Median serum hs-TnT level was significantly elevated among child Pugh class C vs class A (8.6 [IQR: 3.0–18.5] ng/L vs class B 5.2 [IQR 3.0–6.6]; *p* < 0.05) | | 103 |
| Huang CF *et al.* (2010) | Case-control | Mean age: 52.8 ± 10.3  Male: 45%  -  China | HBV, HIV,  autoimmune hepatitis, primary biliary cirrhosis, sclerosing cholangitis, Wilson’s disease, a1-antitrypsin deficiency,  decompensated LC, , hepatic failure, alcohol abuse, psychiatric condition, HCC | hsCRP | | n=190, HCV: 95, Controls: 95  -  - | | -Mean serum hsCRP level significantly higher in HCV vs controls  (0.97 ± 0.11 vs. 0.24 ± 0.07 mg/L; *p<*0.001)  -Serum hsCRP levels significantly decreased in patients after therapy (0.24 vs. 0.62 mg/L; p<0.001 | | 104 |
| Oguz A *et al.* (2013) | Case-control | Mean age: 46.1±6.1  Male: 42.2%  -  Turkey | CHF, CKD, psychosis, pregnancy, hematological disorders, decompensated, LC, and combined hepatitis HCV/HBV or HCV/HBV/HDV | CRP | | n= 75, HCV: 45 Controls: 30  -  - | | -No difference in mean serum CRP levels in HCV vs controls (4.05±2.3 mg/l vs. 3.3±0.6 mg/l; *p* >0.05  -No significant difference in CRP levels between SVR and NR (4.4±0.7 mg/l vs. 5.0±3.7 mg/l ; *p*>0.05) | | 105 |

-: Data not available

ALD: alcoholic liver diseases, ASC HCV: Asymptomatic HCV, CAD: coronary artery disease, CHB: Chronic hepatitis B, CHC: Chronic hepatitis, CHF: congestive heart failure, CLD: Chronic liver diseases, CKD: chronic kidney disease, CRP : C-reactive protein, DM: diabetes mellitus, GIB: gastrointestinal bleeding, HAI: hepatic activity index, HAV: hepatitis A virus, HBV: hepatitis B virus , HCC: hepatocellular carcinoma , HCV: hepatitis C virus, HDV: hepatitis D virus, HIV: human immunodeficiency virus, HR: hazard ration, hsCRP: High-sensitivity C-reactive protein, hs-TnT, high sensitivity troponin T, HTN: hypertension, IFN-α : interferon alpha , IL: Interleukin, IQR: interquartile range, IVDU: intravenous drug use, LC: liver cirrhosis, LPS: lipopolysaccharide, NAFLD: non-alcoholic fatty liver disease, NR: non responders, NT-proBNP: N-terminal pro b-type natriuretic peptide, RA: rheumatoid arthritis, sCD: soluble cluster of differentiation, sE-selectin: Soluble E-selectin, sgp: soluble glycoprotein, sICAM-1: soluble intercellular adhesion molecule-1, sIL-R: soluble interleukin receptor, SLE: systemic lupus erythematosus, sTNFR: soluble TNF-α receptors, sVCAM-1: Soluble vascular cell adhesion molecule-1, SVR : sustained virologic response, TNF-α: tumor necrosis factor alpha, TNFR: TNF-α receptors.

**Supplementary Table 2: Biomarkers of Endothelial Function**

| Study (year) | Design | HCV study population: age in years; sex, %; race/ethnicity; country | Exclusion Criteria | Biomarkers evaluated | Sample Size, follow up time, calendar time | Outcome | Ref. |
| --- | --- | --- | --- | --- | --- | --- | --- |
| Roed T *et al.* (2014) | Case-control | Mean age: 50.8  Male: 61.7%  -  Denmark | Antiviral  therapy, DM, ischemic heart disease, decompensated heart disease, prior cardiovascular event, HIV, HBV, pregnancy, lactation. | sICAM-1  sVCAM-1  sE-selectin  hsCRP | n=120, HCV: 60, Controls: 60  -  2010-2011 | -Mean serum hsCRP level higher in HCV vs. controls (7.15±16.2 mg/ml vs. 3.74±5.1 mg/ml)  -Mean serum sICAM-1 level higher in HCV vs. controls (248.3±137.3 ng/ml vs. 125.5±47.7 ng/ml)  -Mean serum sVCAM-1level higher in HCV vs. controls (1440.8±509.0 ng/ml vs. 1103.6±180.1 ng/ml)  -Mean serum sE-selectin level higher in HCV vs. controls (65.6±41.8 ng/ml vs. 23.4±13.9 ng/ml) | 8 |
| Panasiuk A *et al.* (2004) | Case-control | Mean age: 36  Male: 60%  -  Poland | - | sICAM-1  sP-selectin  IL-6  IL-10 | n=unknown, HCV:40  Controls: not provided  -  - | -Mean sICAM-1 level significantly higher in HCV vs controls (434±104 pg/mL vs 230±29 pg/mL; *p*<0.005  - Mean sICAM-1 levels decreased significantly with treatment in SVR vs NR (*p<*0.0001)  - Mean sP-selectin level significantly higher in NR vs. controls 240 ±101 pg/mL vs 144±75 pg/mL; *p*<0.01)  -Mean serum IL-6 levels significantly higher in SVR vs NR before and during treatment (p*<*0.02)  - Pre-treatment mean serum IL-10 level significantly higher in SVR vs NR (0.88 ±0.78 pg/mL vs 0.45±0.46 pg/mL; *p*<0.05 | 47 |
| Al-Jiffri O (2017) | Case-control | Age range: 28-53  Male: 64%  -  Saudi Arabia | Alcoholism, autoimmune phenomena, or metabolic disorders liver disease | TNF-α  sICAM-1  sVCAM-1  sE-selectin | n= 250, CHC without cirrhosis: 50, CHC with cirrhosis: 50, Controls: 150  -  - | -Mean serum levels of TNF-α, sICAM-1, sVCAM-1, and sE-selectin significantly higher in CHC with and without cirrhosis vs controls (*p*<0.05)  -Mean serum levels of TNF-α, sICAM-1, sVCAM-1, and sE-selectin significantly higher in CHC with cirrhosis vs CHC without cirrhosis (*p*<0.05) | 63 |
| Kaplanski G *et al.* (1997) | Case-control | Mean age: 53  Male: 68.2%  -  France | HIV, HBV | TNF-α  sICAM-1  sVCAM-1 | n= 42, HCV: 22, Controls: 20  -  - | -Mean serum TNF-α level significantly higher in HCV vs. controls (32±7 pg/ml vs. 12±2 pg/ml; *p* < 0.05)  - Mean sICAM-1 and sVCAM-1 levels significantly higher in HCV vs controls (sICAM-1 HCV+ 487 ± 49 ng/ml vs controls 247 ± 20; *p <*0.0001, sVCAM-1 HCV+ 1527±279 ng/ml vs. controls 672 ±50 ng/ml; *p*< 0.01)  -Serum TNF-a levels correlated with sICAM-1 (*r* =0.66; p<0.01)  -sICAM-1 levels correlated with lobular inflammation (*r*=0.8; *p<* 0.01) and sVCAM-1 levels correlated with fibrosis (*r* = 0.7; *p*<0.01) | 64 |
| El-Bassiouni NEI *et al.* (2013) | Case-control | Mean age: 45.6  Male: 63.3%  -  Egypt | Antiviral therapy, anti-coagulants, time CKD, Schistosomiasis infection, chronic viral diseases other than HCV, nonalcoholic steatohepatitis, biliary disorders, malignancies | sICAM-1  sVCAM-1  sE-selectin  TNF-α | n=75, HCV: 60 (CHC:30, LC:30), controls: 15  -  - | -Mean sICAM-1 level significantly higher in HCV with LC vs. CHC and controls and higher in CHC vs. controls (HCV with LC 1027.97±398.18 ng/ml vs. CHC 697.57±429.80 ng/ml, vs. controls 201.53±83.96 ng/ml;  *p*<0.01)  -Mean sVCAM-1 level significantly higher in HCV with LC vs. CHC vs. controls (HCV with LC 1787.31±14.51 ng/ml vs. CHC 1361.33±27.90ng/ml, vs. controls 344.61±29.36 ng; *p*<0.01)  -Mean sE-selectin level significantly higher in HCV with LC and CHC vs. controls (HCV with LC 109.49±50.36 ng/ml, CHC 102.90±47.42 ng/ml vs. controls 42.12±20.26 ng/ml; *p*<0.01)  -Mean TNF-α level significantly higher in HCV with LC vs. CHC vs. controls (HCV with LC 77.43±42.99 pg/ml vs. CHC 36.47±17.81 pg/ml vs. controls 11.73±4.02 pg/ml; *p*<0.01) | 66 |
| Micheloud D *et al.* (2009) | Prospective case-control | Age range: 35-65  Male: 26.3%  -  Spain | Rejection, biliary obstruction, graft injury  . | sVCAM-1 | n= 37 post liver transplant, HCV: 19 (SR: 12, NSR:7), ALD: 18  -  2002-2005 | -Median sVCAM-1 level significantly higher in HCV-SR vs. HCV-NSR and ALD (HCV-SR: 2446.7 ng/ml vs. HCV-NSR: 1266.7 ng/ml and ALD: 1359.1 ng/ml; *p* < 0.01) | 90 |

-: Data not available

ALD: Alcoholic liver disease, CHC: Chronic hepatitis, CKD: Chronic Kidney Disease, CRP: C-reactive protein, DM: diabetes mellitus, HBV: hepatitis B virus, HCV: hepatitis C virus, HIV: human immunodeficiency virus, hsCRP: high-sensitivity C-reactive protein, IL: Interleukin, LC: liver cirrhosis, NSR: non-severe recurrence sCD: soluble cluster of differentiation, sE-selectins: soluble E-selectin, sICAM-1: soluble intercellular adhesion molecule-1, sP-selectins: soluble P-selectin. SR: severe recurrence, sVCAM-1: soluble vascular cell adhesion molecule-1, TNF-α: tumor necrosis factor alpha.

**Supplementary Table 3: Biomarkers of Cardiac Dysfunction**

| Study (year) | Design | HCV study population: age in years; sex, %; race/ethnicity; country | Exclusion Criteria | Biomarkers evaluated | Sample Size, follow up time, calendar time | Outcome | | Ref. |
| --- | --- | --- | --- | --- | --- | --- | --- | --- |
| Antonelli A *et al.* (2012) | Case-control | Mean age: 56  Male: 29.1%  -  Italy | Thyroid disorders, autoimmune diseases,  immunomodulator therapy, HTN, renal  disorders, DM | NT-proBNP, IL-6 | n=110, HCV patients: 55, Controls: 55  -  2001-2006 | | - Mean NT-proBNP level significantly higher in HCV+ vs. controls (120±174 ng/L vs. 17±19 ng/L; *p* < 0.001)    - Mean IL-6 serum levels significantly higher in HCV+ vs controls (54±16 ng/L vs. 11±6 ng/L; *p* =0.005) | 50 |
| Antonelli A *et al.* (2010) | Case-control | Mean age: 55  Male: 35%  -  Italy | LC, HCC, heart failure, CKD  thyroid  disease, DM, malignancy, | NT-proBNP,  TNF-α | n=120, HCV: 60, Controls: 60  -  2001-2006 | | - Mean NT-proBNP level significantly higher in HCV+ vs. controls (162±56 ng/L vs. 31±17 ng/L; *p*< 0.001)  - Mean TNF-α level significantly higher in HCV+ vs. controls (36±74 ng/L vs. 2.1±4.3 pg/L; *p*<0.01) | 67 |
| Che W *et al.* (2012) | Case-control | Mean age: 51.1  Male: 64.2%  -  China | Interferon therapy, LC, liver  disease of different etiology, other infectious diseases,, cardiovascular disease, endocrine diseases, lung disease, CKD,  malignancy, pregnant, postpartum, alcohol or drug abuse. | NT-proBNP,  hsCRP | n=212, HCV: 106, Controls: 106  -  2008-2011 | | - Median NT-proBNP level significantly higher in HCV (64.56 pg/mL (range21.35-145.51)) vs. controls (16.74 pg/mL (range12.43-61.25)); *p* < 0.001  - Median hsCRP level significantly lower in HCV+ (1.023 mg/L (range 0.03-5.379)) vs. controls (3.147 mg/L (range 0.08-7.360); *p*=0.012  -Linear correlation found between hsCRP and NT-proBNP levels among HCV + (*r* = 0.392; *p* = 0.017). | 99 |
| Antonelli A *et al.* (2010) | Case-control | Mean age: 56  Male: 32%  -  Italy | Concomitant liver infection, LC, HCC, autoimmune disease or treated with immune‐modulators. | NT-proBNP | n=100, HCV: 50, Controls: 50  -  2001-2006 | | - Mean NT-proBNP level significantly higher in HCV+ vs. controls (178±175 ng/L vs. 12±16 ng/L; *p* < 0.001) | 125 |
| Okada K *et al.* (2013) | Case-control | Mean age: 58.4  Male: 48.5%  -  Japan | LC, alcohol consumption, HBV, HIV, autoimmune or  drug-induced liver disease, recent antiviral or corticosteroid  therapy, CKD | NT-proBNP | n=198, HCV: 99, Controls: 99  -  2006-2010 | | - Mean NT-proBNP level significantly higher in HCV+ vs. controls (71.6±79.1 pg/mL vs. 39.8±24.4 pg/mL; *p* < 0.05  - Serum HCV RNA levels significantly higher in NT-proBNP > 125 pg/ml group vs <125 pg/ml group; *p* = 0.047  -HCV was independently correlated with NT-proBNP; (OR 18.6 (95% CI 2.40, 144.0); *p*=0.005) | 126 |
| Wang L *et al.* (2011) | Case-control | Age range: 19-50  -  -  China | - | NT-proBNP | n= 867, HCV: 151, HBV: 493, Controls: 223  -  - | | - Median NT-proBNP significantly higher in HCV+ vs. HBV+ and controls (HCV+ 44.76 pg/mL [IQR 24.17- 80.17] vs. HBV+ 35.72 pg/mL [IQR 20.92-71.83] vs. controls 16.46 pg/mL [IQR 12.0-25.9]; *p* < 0.05 | 127 |
| Matsumori A *et al.* (2006) | Retrospective observational cohort | -  -  -  Canada, United States of America | - | NT-proBNP  cTn-I  cTn-T | n=1355 with heart failure, HCV Ab + patients: 59  -  1986-1990 | | - Mean NT-proBNP level significantly higher in HCV+ vs. HCV- (10,000±5860 pg/mL vs. 2508±160 pg/mL; p< 0.001)  -Mean cTn-I and cTn-T levels non-significantly higher in HCV+ vs HCV- (cTn-I 1.9 ± 0.6 vs 1.4 ± 0.2 ng/mL; *p* = 0.2, cTn-T 0.047 ± 0.013 vs 0.039 ± 0.013 ng/mL; *p*= 0.55) | 128 |
| Che W *et al.* (2012) | Case-control | Mean age: 51  Male: 62.2%  -  China | Interferon therapy, LC, liver disease of different etiology, other infectious diseases, cardiovascular disease, endocrine diseases, lung disease, CKD,  malignancy, pregnant, postpartum, alcohol or drug abuse. | NT-proBNP | n=180, HCV: 90, Controls: 90  -  2008-2010 | | - Median proBNP significantly higher in HCV+ vs. controls (62.13 (range 21.04-143.69) pg/mL vs. 17.47 (range 11.73-57.42) pg/mL; *p* < 0.001)  - Impaired diastolic filling on echocardiogram was greater in HCV+ vs. controls (*p*=0.018) | 129 |

-: Data not available

Abbreviations: CHC: Chronic hepatitis, CKD: Chronic Kidney Disease, cTn-I: Cardiac troponin I, cTn-T: Cardiac troponin, DM: diabetes mellitus, HCC: hepatocellular carcinoma, hepatitis B virus, HCV: hepatitis C virus, hsCRP: High-sensitivity C-reactive protein, IL: Interleukin, IQR= interquartile range, LC: liver cirrhosis, MC: mixed cryoglobulinemia, NT-proBNP: N-terminal pro b-type natriuretic peptide, OR= odds ratio

**Supplementary Table 4: Inflammatory and Cardiovascular Biomarkers in HCV/HIV Co-infection**

| Study (year) | Design | HCV study population: age in years; sex, %; race/ethnicity; country | Exclusion Criteria | Biomarkers evaluated; Endpoint | Sample Size, follow up time, calendar time | Outcome | Ref. |
| --- | --- | --- | --- | --- | --- | --- | --- |
| Salter ML *et al.* (2013) | Case-control | Mean age: 47.6  Male: 66.3%  African American: 84.5%  United States of America | HIV monoinfection | CRP  IL-6 | n=1,191, HCV: 703, HCV/HIV: 322, Controls: 166  -  - | -Median serum CRP levels significantly lower in HCV/HIV vs. HCV vs. controls (HCV/HIV: 1.36 [IQR: 0.51-3.88] mg/l vs. HCV: 1.56 [IQR: 0.53-4.82] mg/l vs. controls: 2.13 [IQR: 0.94-5.57] mg/l; *p* = 0.003)  -Median serum IL-6 levels significantly higher in HCV/HIV vs. HCV vs. controls (HCV/HIV: 1.82 [IQR: 1.21-3.15] pg/ml vs. HCV: 1.56 [IQR: 0.98-2.78] pg/ml vs. controls: 1.37 [IQR: 0.90-2.26] pg/ml; *p* = 0.003)  - In MV analysis HCV and HCV/HIV associated with higher mean serum log_e_ IL-6 levels vs. controls (HCV: 0.191, 95% CI 0.043, 0.339 and HCV/HIV: 0.394, 95% CI 0.214, 0.57 vs. controls)  - HCV and HCV/HIV co-infection inversely associated with serum CRP levels: HCV β=−0.523, 95% CI −0.257, −0.789 and HCV/HIV (β=−0.554, 95% CI −0.260, −0.847  -HCV infection consistently associated with decreased serum CRP levels in all strata of liver fibrosis severity (none, mild, severe), albumin, AST or ALT (β=−0.463, 95% CI −0.186, −0.741; *p*<0.05) | 133 |
| Shah S *et al.* (2015) | Case-control | Mean age: 55  Male: 0%  Caucasian: 10%  African American: 90%  United States of America | Older age, high BMI, heavy alcohol use, increased waist circumference. | CRP  IL-6 | n=306, HCV/HIV: 73, HCV: 10, HIV: 164, , Controls: 59  -  2010-2012 | -Median serum CRP level significantly lower in HCV + (HCV/HIV and HCV mono-infected) vs. HIV mono-infected vs controls (HCV: 0.7 mg/dL vs. HIV: 1.2 mg/dL vs. controls: 1.4 mg/dL; *p* = <0.0001)  -Median serum IL-6 level significantly higher in HCV + (HCV/HIV and HCV mono-infected ) vs. HIV mono-infected and controls (HCV/HIV and HCV: 1.5 pg/ml vs. HIV: 0.9 pg/ml and controls: 0.9 pg/ml; *p* = <0.0001)  - For any given IL-6 level, serum CRP levels significantly lower in HCV (HCV/HIV and HCV mono-infected) vs. HIV mono-infected and controls (p<0.001) | 134 |
| Kohli P *et al.* (2016) | Case-control | Mean age: 50  Male: 85%  Caucasian: 52.7%  African American: 39.1%  Latino: 7.3%  Other: 0.9%  United States of America | - | IL-6  hsCRP | n=567, HCV/HIV: 110, HIV: 385 Controls: 72  -  - | -Median serum IL-6 level higher in HCV/HIV vs. HIV vs. controls (HCV/HIV 2.9 pg/ml vs. HIV 2.2 pg/ml vs. controls 1.5 pg/ml)  -No significant difference in median serum hsCRP level in HCV/HIV vs. HIV vs. controls (HCV/HIV 1.3 mg/L [IQR 0.6–3.7] vs. HIV 1.5 mg/L [IQR 0.8–3.5] vs. controls 1.3 mg/L [IQR 0.65–3.1]; *p* = 0.17) | 136 |
| Medrano LM *et al.* (2018) | Case-control | Mean age: 49  Male: 78.6%  -  Spain | HBV, decompensated liver disease, HCC | IL-6  sCD14  sICAM-1  sVCAM-1  sTNFR-1 | n=309, HCV/HIV: 238, HIV: 39, Controls: 32  -  - | -Median serum IL-6 level significantly higher in HCV/HIV vs. HIV and controls (HCV/HIV 5.4 [IQR 3.7-8.1] pg/ml vs. HIV 3.4 [IQR 2.4-4] pg/ml and controls 2.0 [IQR 1.4-3.6] pg/ml; *p* < 0.001)  -Median sCD14 level significantly higher in HCV/HIV vs. HIV and controls (HCV/HIV 5.1 [IQR 3.3-7.4] ug/ml vs. HIV 3.7 [IQR 1.9-5.4] ug/ml and controls: 3.3 [IQR 2.3; 3.9] ug/ml; *p* < 0.01)  -Median sICAM-1 level significantly higher in HCV/HIV vs. HIV and controls (HCV/HIV 2.1 [IQR 1.1-3.9] ug/ml vs. HIV 0.6 [IQR 0.3-1.2] ug/ml and controls 0.4 [IQR 0.1-0.9] ug/ml; *p* < 0.001)  -Median sVCAM-1 level significantly higher in HCV/HIV vs. HIV and controls (HCV/HIV 1.6 [IQR 0.8-3.2] ug/ml vs. HIV 0.3 [IQR 0.2-0.6] ug/ml and controls: 0.3 [IQR 0.2-0.5] ug/ml; *p* < 0.001)  -Median sTNFR-1level significantly higher in HCV/HIV vs. HIV and controls (HCV/HIV: 2.3 [IQR 1.3-3.6] ug/ml vs. HIV: 1.4 [IQR 0.3-2.1] ug/ml and controls: 1.5 [IQR 0.2-2.2] ug/ml; *p* < 0.001)  -Median serum levels of IL-6 and sVCAM-1 significantly higher in HCV/HIV patients with liver stiffness measurement >40 kPa vs. HCV/HIV patients with liver stiffness measurement <12.5 kPa (*p* < 0.001) | 137 |
| de Oca Arjona MM  *et al.* (2011) | Case-control | Mean age: 44  Male: 91.4%  -  Spain | HBV, neoplasia  alcoholic hepatitis, metabolic or autoimmune liver disease, pentoxyfilline, steroidal, nonsteroidal anti-inflammatory, immunosuppressive drugs, recent red blood cell or plasma transfusion | sCD14  TNFR-p55  IL-6 | n=110, HCV/HIV: 70, HIV: 20, Controls: 20  -  2004-2009 | -Median serum sCD14 level significantly higher in decompensated HCV/HIV vs. compensated HCV/HIV and HIV vs. controls (decompensated HCV/HIV 9721 [IQR 8577–10754] ng/ml vs. compensated HCV/HIV 4408 [IQR 2535–5557] ng/ml and HIV 3720 [IQR 3085–3970] ng/ml vs. controls 2941 [IQR 2420–3080] ng/ml; *p <* 0.01)  -Median serum TNFR-p55 level significantly higher in decompensated HCV/HIV vs. compensated HCV/HIV and HIV vs. controls (decompensated HCV/HIV 378 [IQR 246–818] pg/ml vs. compensated HCV/HIV 337 [IQR 201–478] pg/ml and HIV 190 [IQR 131–267] pg/ml vs. controls 149 IQR 103–177] pg/ml; *p <* 0.05)  -Median serum IL-6 level significantly higher in decompensated HCV/HIV vs. compensated HCV/HIV and HIV vs. controls (decompensated HCV/HIV 18 [IQR 10–34] pg/ml vs. compensated HCV/HIV 6 [IQR 4–13] pg/ml and HIV 2 [IQR 1–4] pg/ml vs. controls 1 [IQR 0–2] pg/ml; *p <* 0.01) | 138 |
| Garcia-Broncano P *et al.* (2018) | Cross-sectional | -  Male: 78.6%  -  Spain | HBV, decompensated liver disease, HCC, HIV viral load >50 copies/mL | IL-10  TNF-α | n=277, HCV/HIV: 206, HIV: 39, Controls: 32  -  2012-2016 | -Median serum IL-10 level significantly higher in HCV/HIV vs. HIV and controls ( HCV/HIV 1.4 [IQR 0.8-2.6] pg/ml vs. HIV 0.8 [IQR 0.4-1.7] pg/ml; *p* = 0.003, and controls 0.5 [IQR 0.4-1.5] pg/ml; *p* = 0.001 )    -Median serum TNF-α level not significantly higher in HCV/HIV vs. HIV and controls (2.1 [IQR 0.9-5] pg/ml vs. 1.6 [IQR 0.7-3] pg/ml; *p* = 0.177, and controls 1.5 [IQR 0.7-3.5] pg/ml; *p* = 0.167) | 139 |
| Dong Y *et al.* (2019) | Cross-sectional | Mean Age: 41.2±2.3  Male: 62.5%  -  China | Malignant  neoplasms, acute infection, severe cardiopulmonary, hepatic or renal dysfunction, mental illness, long-term alcohol abuse, HBV, LC pregnancy, breastfeeding | hsCRP  TNF-α | n=80, HCV/HIV: 40, HIV:40  -  2015-2017 | -Mean serum hsCRP level significantly higher in HCV/HIV vs. HIV (35.1±1.6 mg/l vs. 7.9±0.5 mg/l; *p* < 0.001)  -Mean serum TNF-α level significantly higher in HCV/HIV vs. HIV patients (17.4±0.6 ng/ml vs. 8.5±0.3 ng/ml; *p* < 0.001) | 140 |
| Reingold J *et al.* (2008) | Cross-sectional | Male/female  Median Age 41/43  Male: 70.4%  White: 55%/33%  African American 33%/55%  Other: 12%/12%  United States | OIs, <18 years old, pregnant | CRP | n=1135, HCV/HIV 247, HIV: 607, Controls 281  -  - June 2002-September 2002 | - Median serum CRP level lower in HIV/HCV women vs control women (0.92 vs.1.70 mg/L; *p* = 0.012)  - Median serum CRP level similar in HIV/HCV men vs control men (1.01 vs. 0.88 mg/L; *p* = 0.61)  -Co-infection with HCV was associated with 50% lower CRP levels after multivariable adjustment (-50% Effect, (95 % CI -59%, -38%); *p*<0.001) | 141 |
| Floris-Moore M *et al* (2007) | Cross-sectional | Median age: 54  White: 16.7%  Black: 54.2%  Hispanic: 29.2%  Unites States | Myocardial infarction or  stroke | CRP | n= 184, HCV+/HIV+: 110 HCV /HIV+-: 74  -  - | -Median serum CRP level significantly lower in HCV+ vs. HCV - (1.03 vs.1.86 mg/L; p = 0.04)  - In MV analysis, HCV+ men had CRP level 2.47 mg/L lower than HCV- men (R^2^ -039, 95% CI -1.87, 0.29); *p* < 0.001) | 142 |
| Forrester J *et al.* (2009) | Cross-sectional | Mean Age 38/42 (HCV/ HCV/HIV)  Male: 91%/80% ((HCV/ HCV/HIV)  Hispanic: 100%  United States | Pregnancy, non-HIV associated malignancies, hormonal therapy | CRP | n=257, HIV/HCV: 59, HCV: 43, , HIV: 47, controls: 108  -  - | - Median serum CRP levels did not differ significantly in HCV vs HCV/HIV vs HIV vs controls (HCV 2.7 [IQR 0.8-5.1] mg/L vs. HCV/HIV 2.7 [IQR: 0.9-5.4] mg/L vs. HIV 2.6 [IQR: 0.8-6.4] mg/L vs. controls 2.3 [IQR 0.76-5.9] mg/L; *p*= 0.95) | 144 |
| De Castro IF *et al.* (2010) | Cross-sectional | Median Age: 39  Male:74.9%  -  Spain | OIs, drug or alcohol addiction, DM, CKD  autoimmune diseases, hemochromatosis, primary biliary cirrhosis, Wilson’s disease, a1-antitrypsin deficiency, neoplasia | sICAM-1  sVCAM-1 | n=207, HCV/HIV= 183 (SVR= 15), Controls: 24  -  - | -Serum sICAM-1 and sVCAM-1 levels significantly higher in HCV/HIV vs. controls (*p*<0.05)  - Serum sICAM-1 and sVCAM-1 levels significantly higher in HCV genotype 1 vs. non genotype 1 HCV (*p*<0.05)  -On MV analysis, advanced fibrosis associated with serum levels of sICAM-1 (β coefficient 0.165 (SE 0.047), *p*=0.030) and sVCAM-1 (β coefficient 0.190 (SE 0.059), *p*=0.001)  -Patients with SVR had significant decrease in levels of sICAM-1 (SVR *p*=0.001 vs. NR *p=*0.427; difference between SVR and NR groups *p*=0.001) and sVCAM-1 (SVR *p*=0.019 vs. NR *p=*0.053; difference between SVR and NR groups *p*=0.002)  - Change in pattern of these serum markers significantly different between SVR and NR patients (*p*<0.05) | 145 |
| Masiá M *et al.* (2011) | Cross-sectional | Median Age: 43.5  Male:84.1%  -  Spain | Decompensated LC, HCC, malignancy, HBV, hyper- or hypothyroidism, major depression,  drug addiction, active alcoholism, pregnancy, thrombocytopenia | sICAM-1  sVCAM-1 | n= 201 HCV/HV: 63, HIV: 138  -  - | -Median sVCAM-1 level was significantly higher in HCV/HIV vs. HIV patients (sVCAM-1 931.4 [IQR 707.8-1606.5] ng/mL vs. 766.6 [IQR 630.3-974.5] ng/mL; *p*< 0.001)  -Median sICAM-1 level was significantly higher in HCV/HIV vs. HIV patients (492.6 [IQR 354.4-713.1] vs. 314.3 [IQR 251.9-408.3]; *p* < 0.001)  -HCV viral load correlated with levels of sVCAM-1 ( Spearman's rho 0.36; *p* = 0.008) and sICAM-1 (Spearman's rho 0.25; *p*= 0.07)  - No differences in levels of sVCAM-1 or sICAM-1 found based on HCV genotypes (*p>*0.05) | 146 |
| Guzmán-Fulgencio M *et al.* (2011) | Cross-sectional | NR/SVR  Median Age 29/26  Male 85.3%/74.3%  -  Spain | OIs, drug or alcohol addiction, DM, CKD  autoimmune diseases, hemochromatosis, primary biliary cirrhosis, Wilson’s disease, a1-antitrypsin deficiency, neoplasia | sTNF-R1  sE-selectin  sICAM-1  sVCAM-1 | n=116, HCV/HIV: 69 (SVR: 35, NR: 34), HIV 47  -  - | -Median serum sTNF-R1 level significantly higher in HCV/HIV vs. HIV (1.96 (IQR [1.5–2.6] ng/mL vs. 1.03 [IQR 0.5–4.2] ng/mL; *p* < 0.001)  -Median serum sE-selectin level significantly higher in HCV/HIV vs. HIV (223 [IQR 150 –288] ng/mL vs. 118 [IQR 24 –280] ng/mL; *p* < 0.001)  -Median serum sICAM-1 level significantly higher in HCV/HIV vs. HIV (1284 [IQR 1012 –1722] ng/mL vs. 779 [IQR 207 –3133] ng/mL; *p* < 0.001)  -Significant decreases in levels of sE-selectin and sICAM-1 in SVR after 72 weeks of treatment (sE-selectin: 228 [IQR 57.6 –515] ng/ml to 173 [IQR 7.03–501] ng/ml; *p* < 0.001 and sICAM-1: 1289 [IQR 667–3167] ng/ml to 1077 [IQR 318–2346] ng/ml; *p* < 0.001)  -Significant increase in sTNF-R1 levels in NR after 72 weeks of treatment (sTNF-R1: 2.2 [IQR 1.1–4.5] ng/ml vs. 2.1 [IQR 0.6–4.5] ng/ml; *p* < 0.001)  -Significant decrease in levels of sE-selectin and sICAM-1 in NR after 72 weeks of treatment (sE-selectin: 211 [IQR 117 –760] ng/ml vs. 220 [IQR 111–475] ng/ml; *p* < 0.001 and sICAM-1: 1223 [IQR 563–3167] ng/ml vs. 1255 [IQR 254 –3694] ng/ml; *p* < 0.001) | 147 |
| Shaked I *et al.* (2014) | Cross-sectional | Median age: 45  Male: 0%  Black: 62%  Hispanic: 26%  White/Other: 12%  United States | - | sCD14 | n=264, HCV/HIV:66, HCV: 66, HIV: 66, Controls: 66  -  - | -Mean serum sCD14 level was higher in HCV/HIV vs HCV vs HIV vs controls (HCV/HIV 1649 (SD 560) ng/mL vs. HCV 1317 (SD 330) ng/mL vs. HIV 1477 (SD 464) ng/mL vs. 1179 (SD 287) ng/mL  -Mean serum sCD14 level significantly associated with an increased odds of a carotid artery lesion (OR 1.85, 95% CI 1.04, 2.12); *p*=0.03) | 148 |
| Beltran LM *et al.* (2014) | Cross-sectional | Median age: 37.5  Male 76.8%  -  Spain | Cardiovascular disease, DM, CKD, pregnancy, active malignancy, any terminal medical condition | sTWEAK sCD163 | n= 51, HCV/HIV: 3, HIV: 26, Controls: 23 | -Median serum sCD163 level and sCD163/sTWEAK ratio significantly higher in HCV/HIV co-infection vs. non HCV (sCD163 1,290 [IQR 997–2,152] ng/ml vs. 776 [IQR 501–951] ng/ml; *p* = 0.01 and sCD163/sTWEAK 4.08 [IQR 3.04–9.01] vs.2.15 [IQR 1.3–2.71]; *p*<0.001) | 149 |
| Mascia C *et al*. (2017) | Cross-sectional | -  Male 62.8%  -  Italy | Antiviral therapy | sCD163 | N=129, HCV/HIV: 31, HCV: 50 HIV: 21, Controls: 27  -  - | -Median serum sCD163 significantly higher in HCV vs. HIV and control (HCV 1469.6 (range 790.6—3360.3) ng/ml vs. HIV (1469.6 (range 790.6—3360.3) ng/ml vs. Controls 1469.6 (range: 90.6—3360.3; *p=*0.0001) | 150 |
| de Larranaga GF *et al.* (2006) | Cross-sectional | -  Male: 75%  -  Argentina | Pregnancy,< 18 years old , absolute neutrophil count <100 cells/mm^3^, hemoglobin <10 g/dl, coinfection with hepatotropic viruses, LC, pancreatitis,  recent anabolic drug therapy | sVCAM-1 sP-selectin | n=118, HCV/HIV 33, HIV: 85  -  - | -Median serum levels of sVCAM-1 and sP-selectin were significantly higher in HCV/HIV patients on ART vs. HIV patients on ART (sVCAM-1 650 [IQR 463–836] ng/mL vs 482 [IQR 414–601] ng/mL; *p*= 0.003, sP-selectin 78 [IQR 52–101] ng/mL vs 51 [IQR 40–63] ng/mL; *p=* 0.001)  -In MV analysis serum sP-selectin was associated with HCV infection: OR=1.022, 95% CI 0.999, 1.045; *p*=0.0058 | 151 |
| Dabrowska MM *et al.* (2012) | Cross-sectional | Mean age: 40  Male: 81.3%  -  Poland | Alcohol abuse | BNP | n= 80, HCV/HIV-29  HIV= 51  -  - | - Mean serum BNP serum level was significantly higher in HCV/ HIV vs HIV (160.0±130.9 fmol/ vs. 81.9±37.2 fmol/l; *p* < 0.0001) | 152 |

-: Data not available

Abbreviations: ALT: alanine aminotransferase, ART: antiretroviral therapy, AST: aspartate aminotransferase, BMI: body mass index, BNP: brain natriuretic peptide, CI: confidence interval, CHC: Chronic hepatitis, CKD: chronic kidney disease, CRP: C-reactive protein, DM: diabetes mellitus IL: interleukin, IQR: interquartile range, HBV: Hepatitis B virus , HCC: hepatocellular carcinoma, HCV: Hepatitis C virus, HIV: human immunodeficiency virus, hsCRP: high sensitivity C-reactive protein, kPa: kilopascal, LC: liver cirrhosis, MV: multivariate , NR: non responders, OIs: opportunistic infections, sCD :soluble cellular differentiation, SD: standard deviation, SE: standard errors, sE-selectin, soluble E-selectin, sICAM-1: soluble intercellular adhesion molecule-1, sP-selectin: soluble P-selectin, sVCAM-1: soluble vascular cell adhesion molecule-1, SVR: sustained viral response sTNFR: soluble TNF-α receptors, TWEAK: TNF like weak inducer of apoptosis

**Supplementary Table 5: Inflammatory Biomarkers in CHC patients with Other Inflammatory Comorbidities**

| Study (year) | | Design | | HCV study population: age in years; sex, %; race/ethnicity; country | Exclusion Criteria | Biomarkers evaluated; Endpoint | | | Sample Size, follow up time, calendar time | | Outcome | Ref. |
| --- | --- | --- | --- | --- | --- | --- | --- | --- | --- | --- | --- | --- |
| Pimentel JP *et al.* (2013) | Cross-sectional study | | Age range: 18-51  Male: 81.6%  -  Brazil | | - | | IL-6  IL-10  TNF- α | n= 124, HCV+/hemophilia-: 16, HCV+hemophilia+: 22, HCV-/hemophilia+: 46, Controls: 40  -  - | | -HCV patients have higher frequency of high IL-6, IL-10, TNF-α producers vs. low cytokine producers compared to controls | | 45 |
| Antonelli A *et al.* (2010) | Case-control | | Mean age: 58  Male: 27.8%  -  Italy | | Immuno-rheumatic and  neoplastic diseases, heart failure, CKD | | IL-6  NT-proBNP | n= 108, HCV+/cryoglobulinemia+: 54, Controls: 54  -  2001-2006 | | -Mean serum IL-6 significantly higher in HCV with cryoglobulinemia patients vs. controls (63±155 ng/L vs. 5±14 ng/L; *p* = 0.005)  -Mean serum NT-proBNP significantly higher in HCV with cryoglobulinemia patients vs. controls (216±103 ng/L vs. 11±14 ng/L; *p* < 0.001) | | 51 |
| Caliskan Y.  *et al.* (2012) | Case-control | | Mean age: 47  Male: 46.7%  -  Turkey | | HBV, nonrenal cause of anemia other than iron deficiency, recent blood transfusion, malignancy, end-stage liver disease, or chronic hypoxia , recent antibiotics within the past 4 weeks | | hsCRP  TNF-α  IL-6 | n=80, HCV+/ESRD+: 30, HCV-/ERSD+: 30, Controls: 20  -  - | | -Mean serum IL-6 level significantly lower in HCV+/ESRD+ vs. HCV-/ESRD+, both significantly higher than controls (HCV+/ESRD+: 9.1 ± 7.7 pg/ml vs HCV-/ESRD+ 15.8 ± 10.9 pg/ml; p = 0.016 vs. controls 3.5 ± 4.2 pg/ml; *p*= 0.021  -Mean serum TNF-α level non-significantly higher in HCV+/ESRD+ vs. HCV- ESRD+, both significantly higher than controls (HCV+/ESRD+ 76.1 ± 61.0 pg/ml vs. HCV-: 61.0 ± 29.6 pg/ml); *p*= 0.593 vs. controls 6.4 ± 4.6 pg/ml; *p*= 0.026)  -Median serum hsCRP level non significantly higher in HCV+/ESRD+ vs. HCV- ESRD+, both significantly higher than controls (HCV-/ESRD+: 12.20 [IQR 5.07-21.42] mg/L vs. HCV-/ESRD+: 5.45 [IQR 3.25-1.57] mg/L; *p*= 0.181 vs. controls 1.83 [IQR 0.68-2.79] mg/L; *p*= 0.005) | | 55 |
| Cielecka-Kuszyk J *et al.* (2011) | Case-control | | Mean age: 46.6  Male: 41.2%  -  Italy | | Antiviral therapy | | TNF-α  IL-6  IL-10 | n= 55, HCV+/cryoglobulinemia+ 24, HCV/ cryoglobulinemia-: 10, Controls: 21  -  - | | -Median serum TNF-α level significantly higher in HCV+/cryoglobulinemia+ vs. HCV+/cryoglobulinemia - vs. controls (HCV+/cryoglobulinemia+: 2.125 [IQR 1.57-3.873] pg/ml vs. HCV+/cryoglobulinemia-: 1.48 [IQR 1.088-1.96] pg/ml vs. controls 1.02 [IQR 0-1.225] pg/ml; *p <* 0.001)  -Median serum IL-6 level significantly higher in HCV+/cryoglobulinemia+ vs. HCV+/cryoglobulinemia- vs. controls (HCV+/cryoglobulinemia+ 4.315 [IQR 2.263-12.583] pg/ml vs. HCV+/cryoglobulinemia- 1.645 [IQR 1.343-2.6] pg/ml vs. controls 1.04 [IQR 0-1.225] pg/ml; *p <* 0.001)  -Median serum IL-10 level significantly higher in HCV+/cryoglobulinemia+ vs. HCV+/cryoglobulinemia- vs. controls (HCV+/cryoglobulinemia+: 2.995 [IQR 1.98-4.555] pg/ml vs. HCV+/cryoglobulinemia-: 1.59 [IQR 1.31-2.01] pg/ml vs. controls 0 [IQR 0-1.075] pg/ml; *p <* 0.001) | | 56 |
| Mezher MN *et al.* (2017) | Case-control | | -Age range 14-67  - Male: 46.9%  -Iraq | | CKD, other viral infection, anti-viral therapy | | TNF-α | n= 82, HCV+/DM+:14  HCV+/DM-:40, Controls: 18  -  2014-2015 | | -Mean serum TNF-α levels significantly higher in HCV vs. controls (*p* < 0.05)  - Mean serum TNF-α levels non significantly higher in HCV+/DM+ vs. HCV+/DM- (194.4pg/ml vs. 124.6pg/ml; *p*>0.05) | | 68 |
| Elsammak M *et al.* (2005) | Case-control | | Mean age: 52.5  Male: 62%  -  Egypt | | HBV, auto-antibodies, hemochromatosis, Wilson’s disease,  alcohol consumption, amiodarone,  corticosteroids, tamoxifen, methotrexate, oral contraceptives, malignancy, hypo-hyperthyroidism, pregnancy, active infection | | TNF-α | n=90, HCV+/DM+: 23, HCV+/DM-: 27, HCV-/DM+: 22, Controls: 18  -  - | | -Median serum TNF-α level significantly higher in HCV+/DM+ and HCV+/DM- vs. HCV-/DM+ and controls (HCV+/DM+: 19.8 [IQR 0.51–139] pg/ml and HCV+/DM-: 25.5 [IQR 0.43–124.0] pg/ml vs. HCV-/DM+: 0.85 [IQR 0–10.5] pg/ml and controls 0.32 [IQR 0–5.8] pg/ml; *p <* 0.001)  -Significant correlation between HCV RNA load and serum TNF-α (*r=*0.639, *p* =0.01) | | 74 |
| Skowronski M *et al.* (2010) | Cross-sectional | | Mean age: 48.9  Male: 54.3%  -  Poland | | HBV, autoimmune hepatitis, primary biliary cirrhosis, hemochromatosis, Wilson’s disease other liver disease, CKD, LC, chronic inflammatory diseases, anemia,  leucopenia, steroid or nonsteroidal anti-inflammatory drugs | | CRP | n= 208, HCV+/DM-:46, HCV-/DM+: 110, (type 2 DM: 54, type 1 DM: 56), Controls: 52  -  - | | -Median serum CRP level significantly lower in HCV+ vs. DM2 and controls (HCV+ 1.07 [IQR 0.36-2.45] mg/l vs. DM2 2.58 [IQR 1.22- 4.56]; *p* <0.001 vs. controls 0.45 [IQR 0.22-0.90]; *p*= 0.002)    - Median CRP levels non-significantly higher in HCV+ vs. DM1 (1.07 [IQR 0.36-2.45] mg/l vs. 0.91 [IQR 0.42-1.96]; *p*= 0.747) | | 157 |
| Yelken B *et al.* (2009) | Case control | | Mean age: 36  Male: 56.7%  -  Spain | | >60 and/or < 18 years HBV, LC, DM, atherosclerotic disease, CHF , amyloidosis, abnormal electrocardiographic | | hsCRP | n=52, HCV+: 26 HCV-: 26  -  - | | -Mean serum hs-CRP level significantly higher in HCV + vs. HCV- (16 ±14.9 mg/L vs. 7.23 ±5.9 mg/L; *p* =0.012). | | 158 |
| Chennu KK *et al.* (2018) | Cross-sectional | | Mean age: 50.3  Male: 72.7%  -  India | | - | | hsCRP | n= 63, HCV/+ESRD+: 22, HCV-/ESRD+: 22, Controls: 22  -  2010-2011 | | -Mean serum hsCRP level significantly higher in HCV+/ESRD+ vs. HCV-/ESRD+ vs. controls (HCV+/ESRD+: 8.66±5.41 mg/dL vs. 3.85±1.77 mg/dL vs. 0.1200±0.06 mg/dL; *p*<0.001) | | 159 |
| Pawlak K *et al.* (2007) | Cross-sectional | | Mean age: 58 ± 12  Male: 56.3%  -  Poland | | Inflammatory and infectious  diseases, malignancies, DM, autoimmune diseases, active liver diseases, cryoglobulinemia or vasculitis | | hsCRP  TNF-α | n=48, Hepatitis+/ESRD+: 14 (HCV:12, HBV: 4 HCV/HBV: 3) Hepatitis-/ESRD+: 16, Controls: 18  -  - | | -Mean serum hsCRP level significantly higher in Hepatitis+/ESRD+ vs. controls (Hepatitis+/ESRD+ 6.19 (SD 0.14–68.00) μg/ml vs. controls 0.79 (SD 0.1–10.9) μg/ml; *p*<0.001)  -Mean serum TNF-α level significantly higher in Hepatitis+/ESRD+ vs. Hepatitis-/ESRD+ and controls (Hepatitis+/ESRD+ 3.5 (SD 0.06–7.0) pg/ml vs. hepatitis-/ESRD+ 1.0 (SD 0.06–7.0) pg/ml and controls 0.06 (SD 0.06–0.5) pg/ml; *p*<0.001) | | 160 |
| Caliskan Y *et al.* (2009) | Prospective cohort | | Age range: 25–67  Male: 13  –  Turkey | | HBV, impaired blood glucose level, family history of DM, medication known to affect glucose tolerance or insulin secretion, abnormal thyroid tests, gastrectomy, chronic pancreatitis | | hsCRP | n=72, HCV: 36, Controls: 36  59 months  – | | - Mean serum hsCRP non significantly lower in HCV+ vs. HCV- (1.43 ± 1.93 mg/dL vs. 1.08 ± 1.17 mg/dL; *p*>0.05) | | 161 |
| Nascimento MM *et al.* (2005) | Cross-sectional | | Median: 47 years  Males: 47.5%  -  Brazil | | Chronic inflammatory disease, active infection, HBV. | | hsCRP  IL-6 | n= 140, HCV+/ESRD+: 62, HCV-/ESRD+ :56 Controls: 22  -  - | | -Median serum hsCRP level non significantly lower in HCV+ vs. HCV- (6.6 (range 0.2-62) mg/L vs. 11.8 (range 0.3-150) mg/L; *p*>0.05)  -Median serum IL-6 level non significantly higher in HCV+ vs. HCV- (4.7 (range 0.9-19) pg/ml vs. 42 (range 0.98 -19.64); *p*<0.05)  - Median serum hsCRP/IL-6 ratio significantly lower in HCV+ vs. HCV- (0.7 vs 0.9; *p* < 0.05) | | 162 |
| Afzal N *et al.*  (2011) | Cross-sectional | | Male: 39.5%  Age range: 21-65  -  India | | Chronic inflammatory  disease, autoimmune disorder, acute infection,  malignancy, pregnancy,  HBV, HIV | | IL-6  CRP | n= 43 , HCV+ /ESRD+: 24, HCV- /ESRD+: 19  -  - | | -Mean serum IL-6 level non significantly higher in HCV+ vs. HCV- (*p*>0.05)  -Mean serum CRP level non significantly lower in HCV+ vs. HCV- (*p*>0.05) | | 163 |
| Falasca K *et al.* (2007) | Case-control | | Mean age: 57.3/55.5 (MC+/MC-)  Male: 60%  -  Italy | |  | | IL-6 | n=80, HCV+/MC+: 30, HCV+/MC-: 30, Controls: 20  -  - | | - Mean serum IL-6 level significantly higher in HCV+/MC+ vs. HCV+/MC- (8.7± 4.5 pg/mL vs. 4.6 ± 2.3 pg/mL; *p*<0.0001) | | 164 |
| el-Din HM *et al.* (2004) | Case-control | | Mean age: 39.1  Male: 50%  -  Egypt | |  | | IL-6  sTNFR | n= 80, NHL+/HCV+: 20, NHL+/HCV-: 20, NHL-/HCV +: 20, Controls: 20  -  - | | -Mean serum IL-6 levels similar in NHC+/HCV+ vs. NHC+/HCV- vs. NHL-/HCV+ vs. NCC HCV- (NHC+/HCV: 31 (SD ±34) pg/ml vs. NHC+/HCV-: 25 (SD ±36) pg/ml vs. NHL-/HCV+:14 (SD ±9) pg/ml vs. controls: 15 (SD ±12) pg/ml; *p* =0.49)  -Mean sTNFR levels significantly higher in NHL HCV+ vs. NCC HCV- (2.9 (SD ±1.7) ng/ml vs. 1.9 (SD±2.2) ng/ml; *p* <0.001) | | 165 |
| Siloşi I *et al.*  (2017) | Cross-sectional | | Mean Age: 54.2  Male: 73.2%  -  Romania | | HIV, HBV, other hepatotropic virus co-infection, psoriatic arthritis, SLE, Sjögren’s syndrome, dermatomyositis, mixed connective tissue disease | | IL-6,  TNF-α | n=82, HCVrA:24, RA:30 , Controls: 28  -  - | | -Mean serum IL-6 level significantly higher in HCV+ vs. controls (29.13 pg/mL, 95% CI: 20.01, 38.24 vs. 2.85 pg/mL, 95% CI: 2.08, 3.62; *p*<0.001)    -Means serum TNF-α level significantly higher in HCV+ vs. controls (54.63 pg/mL, 95% CI 36.50, 72.76 vs. 4.29 pg/mL, 95% CI 3.33, 5.25); *p* < 0.0001 | | 166 |
| Ramos-Casals M *et al.* (2002) | Cross-sectional | | Mean age: 66  Male: 90%  -  Spain | | - | | IL-6  IL-10  TNF- α | n= 67, HCV+/SS+: 20 HCV-/SS+: 47  -  - | | -Mean serum IL-6 level significantly higher in HCV+ vs. HCV- (73.6 (SEM ± 47) pg/mL vs. 33.0 (SEM ± 11) pg/mL; *p*=0.045)  -Mean serum IL-10 level significantly higher in HCV+ vs. HCV- (6.7 (SEM ± 1) pg/mL vs. 3.1 (SEM ± 0.6) pg/mL; *p*=0.01),  - Mean serum TNF-α level significantly higher in HCV+ vs. HCV- (59.8 (SEM ± 13) pg/mL vs. 31.7 (SEM ± 3) pg/mL; *p=*0.003). | | 167 |
| Antonelli A *et al.* (2008) | Case-control | | Mean age: 62  Male: 21.6%  -  Italy | | Immune, rheumatic and neoplastic diseases | | TNF- α | n= 306, HCV+/MC+: 102, HCV+/MC-:102, Controls: 102  -  1999-2005 | | -Median serum TNF- α level significantly higher in HCV+/MC+ vs. HCV+/MC- vs. controls (HCV+/MC+ 12.0 [IQR 9.8] pg/mL vs. HCV-/MC+ 5.7 [IQR 5.4] pg/mL; *p* = 0.0002 vs. controls 1.3 [IQR 2.1] pg/mL; *p* < 0.0001) | | 168 |
| Riccio A *et al.* (2012) | Cross-sectional | | Mean age: 46  Male: 85.7%  -  Italy | |  | | IL-6,  sIL-6R sgpl30. | n=65, HCVrA: 21, RA: 24, Controls: 20  -  - | | -Median serum IL-6 level significantly higher in HCVrA and RA vs. controls (HCVrA 60.5 (range 3.6-246.7) pg/ml, RA 54.9 (range 17.5-124.3) pg/ml vs. controls: 20.0 (range 14.1-46.2) ng/ml; *p*=0.001) | | 169 |
| Realdon S *et al.* (2001) | Cross-sectional | | -  Male:51.7%  -  Italy | |  | | sTNF-RI/II | n=149, HCV+:120 (HCV+/LD+:60, HCV+/LD-:60) HCV-/LD+: 19, Controls: 10  -  - | | -Mean serum levels of sTNF-R I and sTNF-R II significantly higher in HCV+ vs. controls (sTNF-RI, 1274 (SD ± 315) pg/ml vs. 903 (SD ± 353) pg/ml, *p=* 0:05; sTNF-RII, 3583 (SD ± 745) pg/ml vs. 2176 (SD ± 340) pg/ml, *p*=0:005).  - Mean serum levels of sTNF-R I and sTNF-R II similar between HCV+ and HCV- LD (sTNF-RI, HCV+ 1410 (SD ± 815) pg/ml vs. HCV-1135 (SD ± 630) pg/ml, sTNF-RII HCV+ 3550 (SD ± 416) pg/ml vs. HCV- 2773 (SD ± 1214) pg/ml; *p*>0.05)  -Highest levels of sTNF-R I and sTNF-R II in HCV+ (vs. HCV- and controls) with type II cryoglobulinemia and lymphoplasmacytoid lymphomas (*p*= 0.01) | | 170 |

-: Data not available

Abbreviations: CHF: congestive heart failure, CKD: chronic renal diseases, CRP: C-reactive protein, DM: diabetes mellitus, DM1: type 1 DM, DM2: type 2 DM, ESRD: end stage renal disease, HBV: hepatitis B virus, HCV: hepatitis C virus, HCVrA : HCV related arthritis, HIV: human immunodeficiency virus, hsCRP: High-sensitivity C-reactive protein, IL: interleukin, LD: lymphoproliferative disorder, MC: mixed cryoglobulinemia, NHC: Non-Hodgkin’s lymphoma NT-proBNP: N-terminal pro b-type natriuretic peptide, RA: rheumatoid arthritis, SEM: standard error of mean, SD: standard deviation, SLE: systemic lupus erythematosus, SS: Sjögren's syndrome, sTNF-R: soluble tumor necrosis factor receptor, TNF: tumor necrosis factor.
