# Supplementary material for: Inflammatory and cardiovascular diseases biomarkers in chronic hepatitis C virus infection: A review
Source: Clin Cardiol. 2019 Nov 30;43(3):222–34. doi: 10.1002/clc.23299 (PMC7068107; doi:10.1002/clc.23299)
Supplement: Supplementary file 2 — FIGURE S1 Prisma flow diagram [file CLC-43-222-s002.docx]

**Supplementary Figure 1: PRISMA Flow Diagram**

Full-text articles excluded (n=147):

Lack of HCV-negative controls (n=66), biomarkers of interest not included (n=39), studies of biomarker expression without serum levels (n=27), relevant data missing (n=8), conducted in a pediatric population (n=4), only included serum levels of stimulated cytokines (n=3)

Records excluded by review of title and abstracts
(n = 2156)

Additional records identified through ancestry and bibliography searches of all relevant articles sources
(n = 24)

Studies included in qualitative synthesis
(n = 115)

Full-text articles assessed for eligibility
(n = 262)

Records screened
(n =2418)

Records after duplicates removed
(n = 2418)

Records identified through database searches
(n = 2430)
